# Doped Graphene Quantum Dots UV–vis Absorption Spectrum: A High-Throughput TDDFT Study

**DOI:** 10.1021/acsomega.2c06091

**Published:** 2023-01-05

**Authors:** Şener Özönder, Caner Ünlü, Cihat Güleryüz, Levent Trabzon

**Affiliations:** † Institute for Data Science & Artificial Intelligence, Boğaziçi University, Istanbul 34342, Turkey; ‡ Department of Chemistry, Istanbul Technical University, Istanbul 34469, Turkey; ¶ Department of Physics, Marmara University, Istanbul 34722, Turkey; § Department of Opticianry, Altınbaş University, Istanbul 34217, Turkey; ∥ Department of Mechanical Engineering, Istanbul Technical University, Istanbul 34469, Turkey

## Abstract

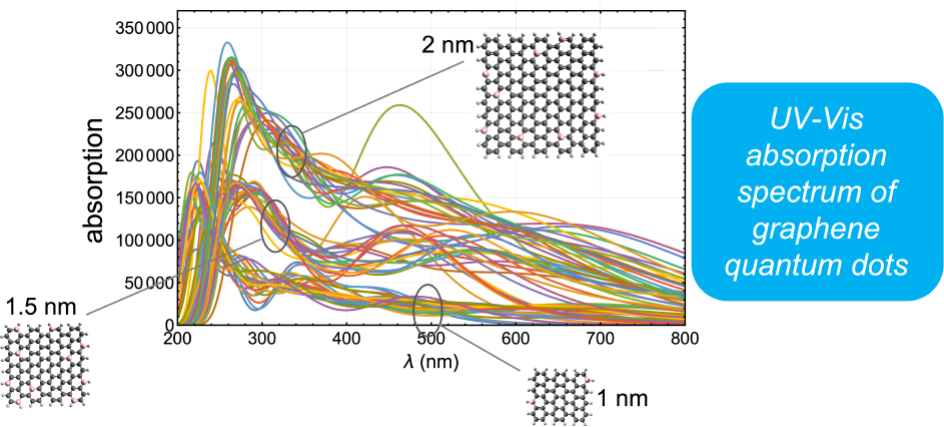

We report on time-dependent density functional theory calculations of the excited states of 63 different graphene quantum dots (GQDs) in square shape with side lengths of 1, 1.5, and 2 nm. We investigate the systematics and trends in the UV–vis absorption spectra of these GQDs, which are doped with elements B, N, O, S, and P at dopant percentages of 1.5%, 3%, 5%, and 7%. The results show how the peaks in the UV and visible parts of the spectrum as well as the total absorption evolve in the chemical parameter space along the coordinates of size, dopant type, and dopant percentage. The absorption spectra calculated here can be used to obtain particular GQD mixture proportions that would yield a desired absorption profile such as flat absorption across the whole visible spectrum or one that is locally peaked around a chosen wavelength.

## Introduction

Graphene quantum dots (GQD) are two-dimensional, a few nanometer-sized nanocrystals with tunable optical properties. Their applications are ranging from solar cells to semiconductors as well as energy storage and biomedical research.^1−3^ GQDs offer great functionality for light-harvesting and photoluminescence applications since their optical properties can be tuned by changing their size and chemically doping them with different elements. GQDs also inherit all other useful properties of graphene such as low toxicity, low cost, and easy production and biocompatibility.

The absorption spectrum of graphene determines its light-harvesting capacity and depends on the underlying electronic structure. The infinite graphene crystal is a zero-band gap semimetal, but when it is reduced to a nanocrystal of a few nanometers, quantum confinement effects set in and a band gap emerges. Also, chemical doping alters graphene’s electronic structure and turns it into a p-type or n-type semiconductor depending on the dopant type.^4^ In the applications of solar cells, quantum dot labeling, quantum dot enhanced photosynthesis, and optical sensors, the absorption spectrum of the material needs to be engineered in order for it to be sensitive to the targeted part of the spectrum.

Absorption spectra of selected GDQs with a particular size, shape, and dopant type have been investigated in the past; however, a complete systematic study considering the full chemical space of GDQs is currently lacking. Here we report on a high-throughput scanning of GQDs of size 1–2 nm of dopant elements B, N, O, S, and P and of dopant percentages from 0% to 7% via time-dependent density functional theory (TDDFT) calculations. The goal of this work is to chart the GQD chemical landscape and to extract the physics on the systematics of how absorption spectrum depends on the nanocrystal size and also how it is altered with chemical doping. This information is necessary for a better spectrum engineering of GQDs.

GQDs have been used in solar cells as an additive to better utilize the UV part of the spectrum that is otherwise left unharvested in conventional solar cells. Typically, GQDs possess n−π and n−π* absorption bands which are located in the UV region of the light spectrum. The absorption bands of GQDs can be controlled by manipulating either the size or composition of GQDs through doping with heteroatoms. Graphene is an excellent electron acceptor with mobility around 7 × 10^4^ cm^2^·V^–1^·s^–1^ and therefore has a great potential to improve efficiency of solar cells as a charge carrier.^5^ GQDs, very small-sized graphene fragments whose band gap can be controlled, are already in use for improving photovoltaic parameters of solar cells (photoconversion efficiency, the peak power, the short-circuit current density, the open circuit voltage, and the fill factor).^5,6^

In principle, GQDs can be produced through top-down or bottom-up synthesis methods.^7,8^ However, controlling the quality and quantity of the dopant and the size of a GQD can be achieved through bottom-up synthesis methods more precisely.^8−10^ GQDs can be synthesized through several different bottom-up synthesis methods such as the hydrothermal synthesis method, microwave assisted synthesis method, and solvothermal synthesis method.^8^ Each bottom-up technique depends on incomplete carbonization of a suitable carbon precursor, and generally, the carbon precursor is chosen among biocompatible and easily affordable ones like citric acid, glucose, etc. The size control of GQDs can be achieved by controlling synthesis parameters such as temperature, pressure, carbon precursors, and solvent. Also, the composition of GQDs can be controlled by addition of an extra heteroatom precursor (N, B, S, and P). As a result, the optical parameters of GQDs can be manipulated via bottom-up synthesis techniques by controlling the synthesis conditions and carbon or heteroatom precursors.^8^

GQDs come in different shapes and dopant content and their absorption and emission properties are determined by these structural properties. It is often not possible to estimate the optical properties of GQDs from their molecular configurations by simple heuristic means, nor is it possible to explore the optical properties of vast number of GQD derivatives through laboratory synthesis. Also, prior knowledge on the structure of a compound is needed to guide a chemist in what to try in the lab. Density functional theory (DFT) provides such guidance where vibrational, structural, electronic, and optical properties of a molecule or crystal can be calculated *in silico*. In addition, computational chemistry methods shed light into the underlying physical mechanisms as well as possible effects that can be understood only via simulations and are otherwise likely to be missed due to environmental effects and errors in the measurement processes. Particularly for exploring the optical properties such as absorptance and fluorescence of molecules and crystals at a reasonable cost, time-dependent density functional theory (TDDFT) has become the gold standard in recent years.^11−15^ TDDFT can be used to calculate the excited states from which absorption and emission spectra can be calculated, and it is used for discovering and designing new compounds and as a complementary source of information for verification and interpretation of the experimental results.

There are several TDDFT studies in the literature that provide excited states and absorption spectra of GQDs of particular size and dopant content. Some of those past work focus on GDQs in specific shapes such as triangle or hexagonal while some others focus on different percentages of a single dopant element.^16−27^ These individual studies do not adequately capture the mapping between the various possible GQD structures and their absorption spectra. In this work, we aim to fill this gap by calculating absorption spectra of 63 different GQDs in the 3D parameter space (i) for side length of 1, 1.5, and 2 nm, (ii) for dopants elements B, N, O, S and P, and (iii) for dopant percentages 0%, 1.5%, 3%, 5%, and 7%. The results can be used to find out the particular GDQ or mixture of them which will absorb the part of the spectrum the most as required by the specific application in use.

## Computational Details

**Figure 1:**
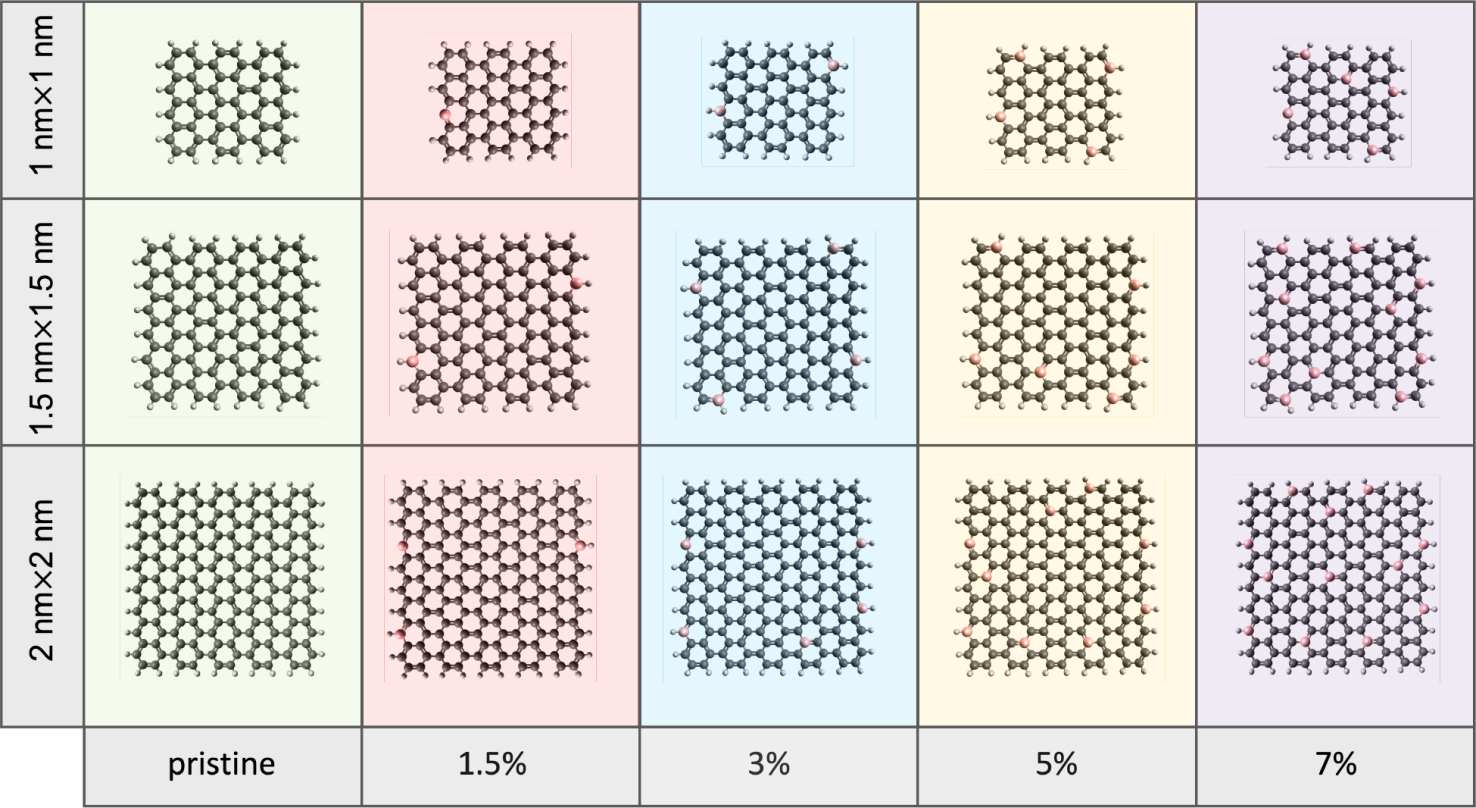
Topology of the GQDs whose excited states and UV–vis spectra are calculated. The carbon atoms are shown in gray whereas the dopant elements are shown in pink. The circumferences are passivized with hydrogen atoms. Pristine 1, 1.5, and 2 nm GQDs (leftmost column) are C_54_H_20_, C_104_H_28_, and C_170_H_36_, respectively.

We calculate square-shaped graphene nanosheets with side lengths 1, 1.5, and 2 nm. The carbon atoms on the perimeter are passivized with hydrogen atoms. In some cases, additional hydrogen atoms are added depending on the dopant element type and percentage, in order to saturate the free bonds and consequently ensure that the nanocrystal is charge neutral and in an *S* = 1 singlet spin state. Figure 1 shows dopant locations for different dopant percentages for all three sizes.

Both geometry optimization and excited-state calculations of GQDs have been performed with Gaussian16 by using the hybrid functional B3LYP with the basis set 6-31G(d). Past studies show that the model B3LYP/6-31G(d) strikes the best balance between computational cost and accuracy.^14,20,28−30^

Water was chosen as the solvent and incorporated in the calculations via a polarizable continuum model (PCM) during both geometry optimization and excited-state calculations. During the optimization evaluations, we also calculated the vibrational frequencies to ensure the system is truly at the minimum of the potential energy surface. In cases where the optimization ended up with negative frequencies, we slightly distorted the atomic configuration in the direction of those negative frequency vectors and reran the optimization until no negative frequencies remained. Once the optimized geometries were obtained, vertical electronic excitation energies of each GQD were calculated with the TDDFT method for the UV–visible part of the spectrum, i.e., 1.6–5 eV (775–248 nm). For the hybrid functional B3LYP used, the errors in the excited-state energies are expected to be in the range of 0.20–0.25 eV.^13,14^

## Results and Discussion

**Figure 2:**
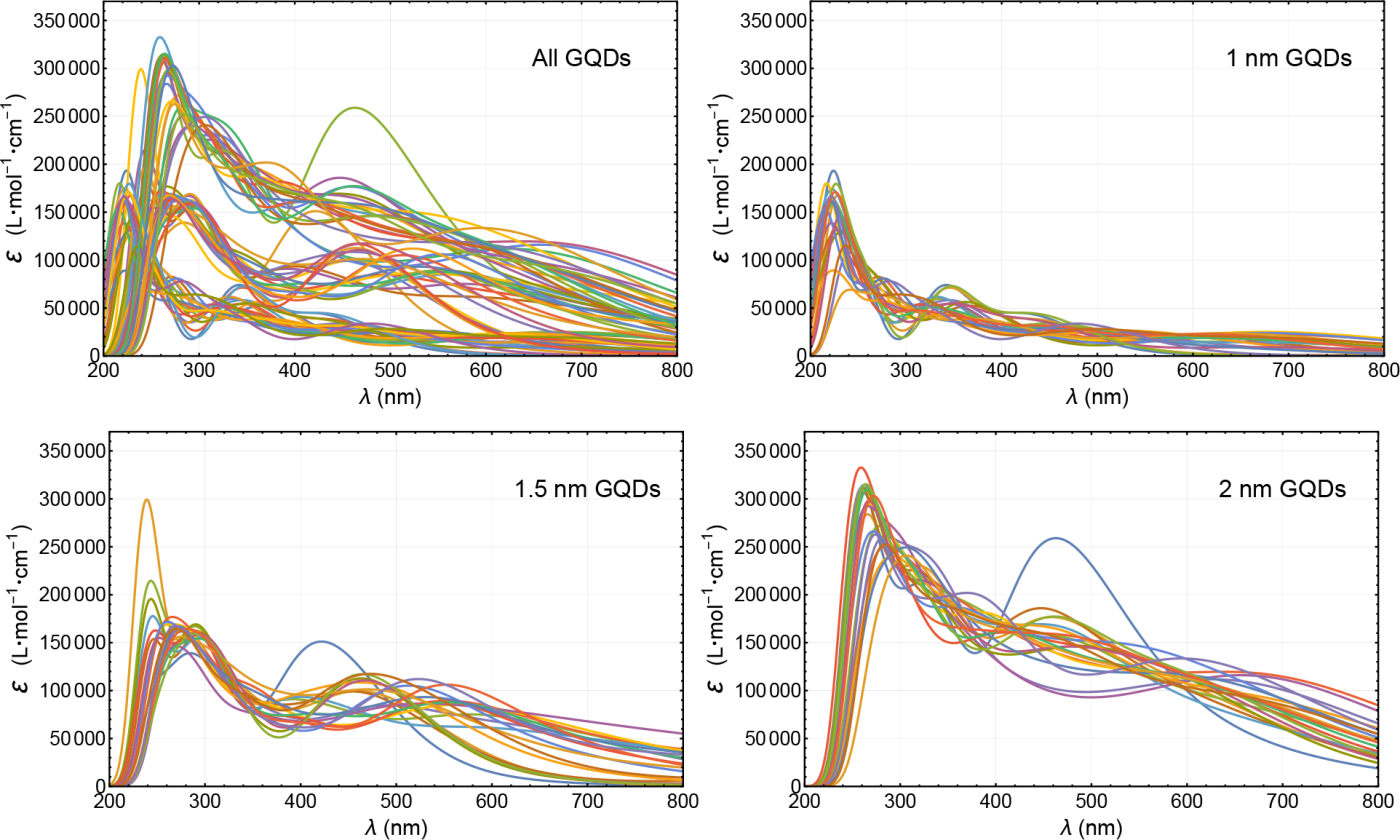
UV–vis absorption spectra of GQDs. Top-left panel includes the spectra of all 63 different GQDs, and the other panels have GQDs grouped with respect to their sizes (side lengths). These spectra are calculated with TDDFT with solvent being water. The size-dependent trends are visible in the figures. The spectrum of each GQD with the labels of size, dopant type, and dopant percentage are provided separately in the Supporting Information.

The size, dopant type, and dopant percentage change the electronic structure of the GQDs; hence, their absorption spectrum changes accordingly. We convolved transition energies with Gaussian distributions using an fwhm of σ = 0.4 eV and used the oscillator strengths to calculate the absorption spectrum for each GQD. Figure 2 presents the absorption spectra of 63 GDQs plotted by using the TDDFT excited-state calculations in this work. The individual absorption spectra of each GQDs are given in the Supporting Information (SI). It is visible from the plots that the absorption spectrum, both the magnitude and profile, depends on size, dopant type, and dopant percentage.

In Figure 2, the spectrum in the bottom left panel with a second peak around 420 nm and the spectrum in the bottom right panel with a second peak around 480 nm correspond to 1.5 and 2 nm pristine (undoped) GQDs. Even though these two spectra seem to be outliers, they have relatively enhanced absorption in 400–500 nm most likely because they do not contain a foreign dopant element to break the high symmetry of the carbon hexagonal structure where certain electronic conjugations are allowed to enhance the absorption.

**Figure 3:**
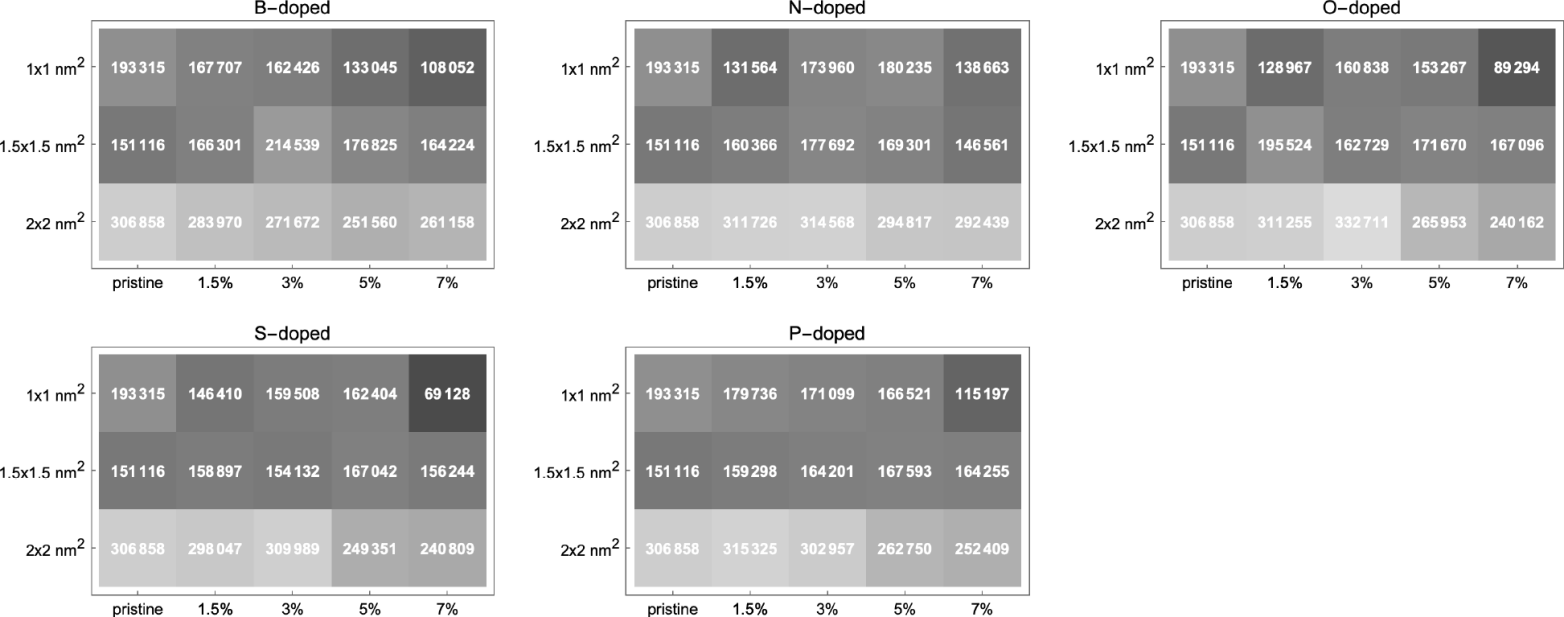
Peak values of extinction coefficient of pristine and doped GQDs calculated via TDDFT. The labels at the top of each plot refer to the dopant elements. For each dopant, a separate array plot with axes of size and dopant percentage is given, and pristine GQDs are added to each plot for comparison. The peak values of the extinction coefficients given in each cell are usually positioned in the UV region with a few exceptions, which can be examined in detail from the individual spectra given in the Supporting Information. The overall trend is that the magnitude of the absorption (extinction coefficient) peak value increases with increasing graphene size while it decreases with increasing dopant percentage.

The spectra turn out to be grouped in three different “size bands” with characteristic profiles as seen in Figure 2. Within a given size band, there is a trend that the magnitude of the absorption decreases with increasing dopant percentage. This is more apparent in the array plots given in Figure 3. On the other hand, absorption (extinction coefficient) increases with increasing GQD size. These trends look similar for all five dopant elements used (B, N, O, S, and P).

**Figure 4:**
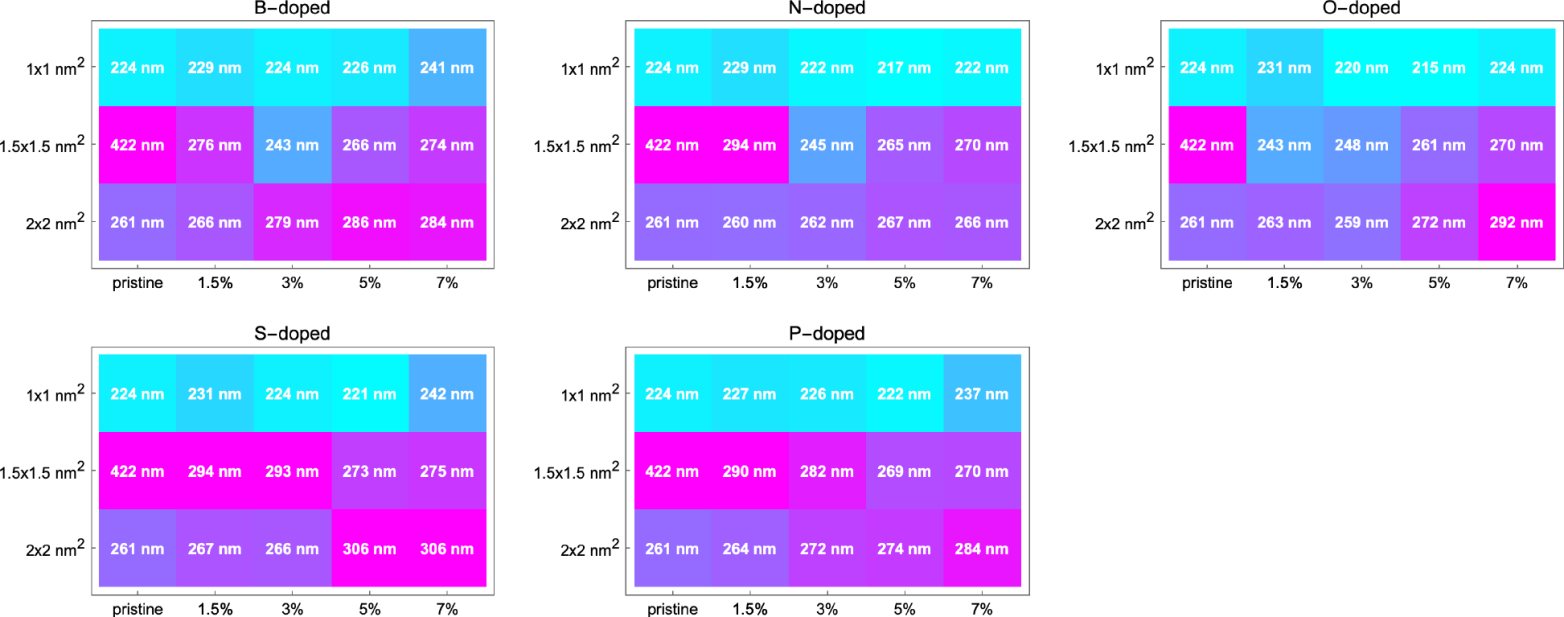
Positions of the absorption peaks in wavelength for each GQDs in the UV–vis region (200–800 nm) as calculated via TDDFT. The letters refer to the dopant elements. Generally speaking, red-shifting of the absorption peak is observed with increasing size as well as increasing dopant percentage.

Figure 4 shows the positions of the absorption peaks in wavelength. In most cases, the peaks are red-shifted with increasing graphene size. This effect can be understood through the idea that larger size allows excitations with longer wavelengths.

**Figure 5:**
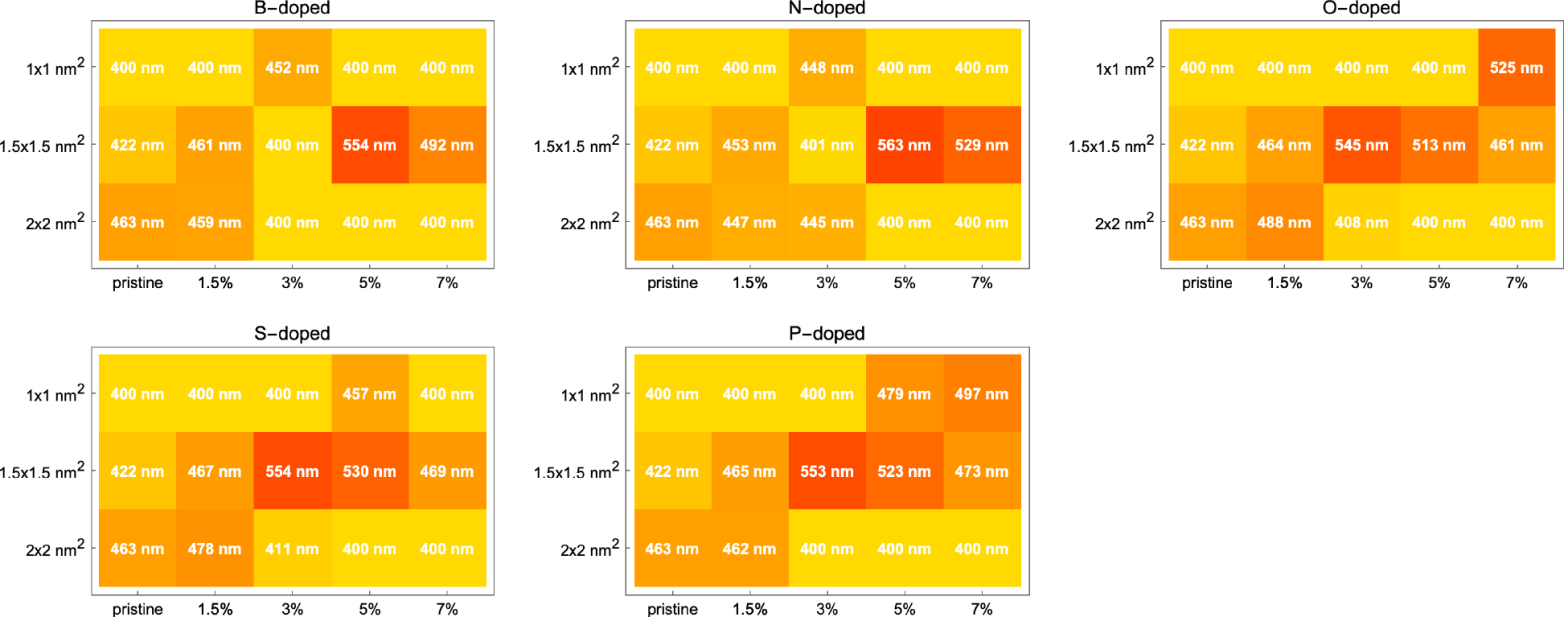
Positions of the absorption peaks in wavelength for each GQDs in the visible region (λ > 400 nm) as calculated via TDDFT. The letters refer to the dopant elements. Relative red-shifting seems to be occurring for the GQDs corresponding to the cells in the middle of each array plot.

The analysis of the peak positions can be narrowed down to only the visible region if only the absorption of visible light is of interest in a given application. Figure 5 shows the position of the absorption peaks in wavelength in the visible region (λ > 400 nm).

**Figure 6:**
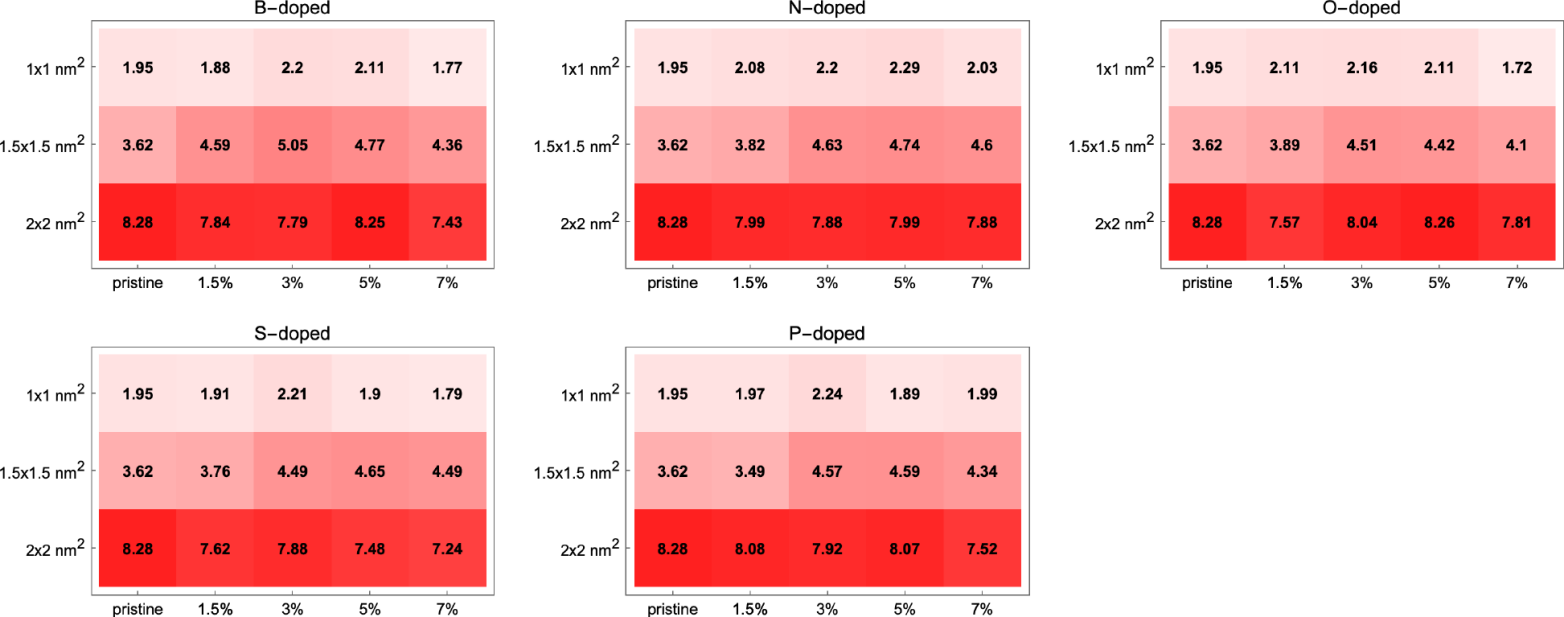
Total amount of absorption in the whole UV–vis spectrum in units of L · mol^–1^ found from the integral of the absorption spectra that were calculated via TDDFT. Total absorption increases with increasing GQD size. The letters refer to the dopant elements. Total absorption grows with increasing GQD size. The change with dopant type and dopant percentage exists but the latter does not have a simple trend common to all cases.

So far, we’ve presented the systematics on how the absorption peak values and their positions in the wavelength change depending on size, dopant type, and dopant percentage. For certain applications, the matter of interest may not be the absorption profiles but the total amount of absorption in the whole UV–vis spectrum. Figure 6 shows the integral of the absorption curves in units of L·mol^–1^. The results show that the total absorption over the whole UV–vis spectrum increases with increasing GQD size. It also changes with dopant type and dopant percentage, but the direction of change varies depending on the dopant and GQD size, so this should be examined case by case from the array plots.

The results above demonstrate that the conjugated π-system of the larger graphene nanosheets enable them to harvest light at longer wavelengths which are missed by the smaller nanosheets most likely due to quantum confinement effects in smaller ones. Thus, an overall red shift in the absorption spectrum with increasing GQD size is observed in the results. Similarly, larger graphene nanosheets have a higher total absorption since for them conjugations at both longer and shorter wavelengths are available. The dopants disrupt this conjugated π-system and reduce the absorption at a given frequency in comparison to that of the pristine graphene; however, the effect of dopant percentage on the absorption profile is not drastic. Variation in the dopant element type creates slight changes in the absorption profile too, but the main determiner of the bands given in Figure 2 is still the nanosheet size. Nevertheless, it should be noted that the effect of dopant on optical parameters becomes more significant for larger GQDs (above 1 nm × 1 nm). So, the theoretical results showed that doping GQDs with heteroatoms can be very useful to manipulate optical parameters when size control is not possible and GQDs with size around 1 nm × 1 nm cannot be obtained.

Solvent effects on graphene quantum dots were studied extensively in ref (31). The findings for C_54_H_18_ and C_54_H_22_ GQDs show that the UV-absorption spectrum peak height and position are practically the same for the solvents water, ethanol, DMF, and acetic acid whereas the no-solvent case (gas phase) is blue-shifted for 10 nm with the peak height reduced to 65% relative to the cases with solvents. Similar solvent effects have also been observed in the doped GQD cases.

For the visible region (λ > 400 nm), the photophysical properties of B- and N-doped GQDs have been studied experimentally in detail. Experimental data found in the literature is in complete agreement with the calculated spectra in this study. The fluorescence emission peak of carbon dots, which are doped either with B or N, shifts to the blue-light region compared to that of pristine GQDs.^32,33^ As the boron amount was increased in N- and B-doped GQDs, the excitation spectrum peaks of GQDs in the visible region (∼400 nm) blue-shifted, which is totally in agreement with the calculated spectra here.^9^ It should be noted that there has been no comparative study for S-, P-, or O-doped GQDs with pristine GQDs in terms of photophysical properties.

**Figure 7:**
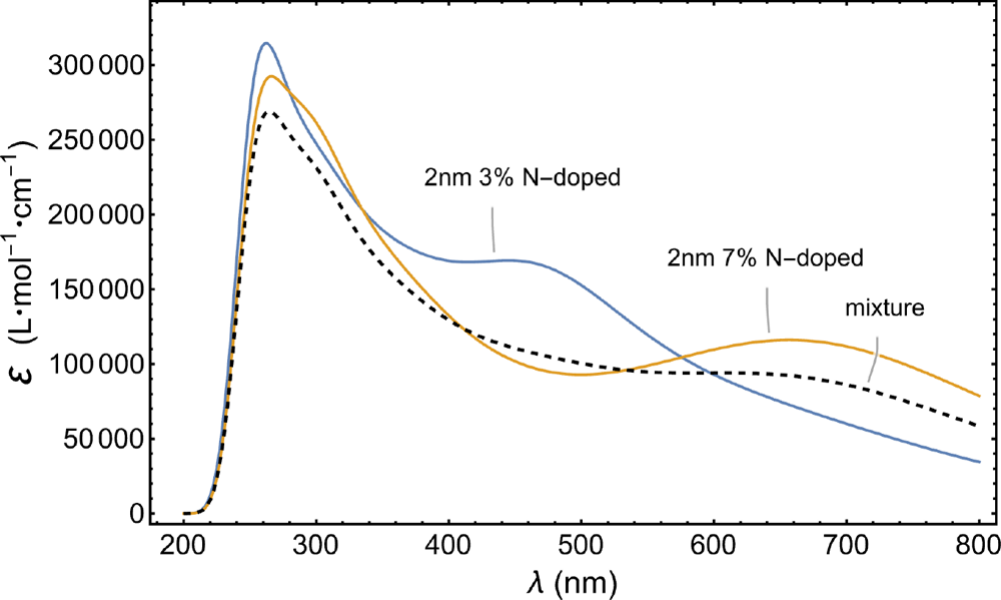
Absorption spectrum of the mixture, shown with a dashed line, of two different GQDs producing a fairly flat absorption profile in the visible spectrum (400–750 nm). The mixture here contains 28% from “2 nm 3% N-doped GQD” and 62% from “2 nm 7% N-doped GQD”.

From the point of application, the outcome of this work can be used for spectrum engineering for the situations where certain parts of the UV–visible spectrum may be desired to have relatively more absorption. For example, in the case of solar cell applications, one may be interested in a specific mixture of the GQDs that has an absorption profile as close as to a flat one across the whole visible spectrum. Figure 7 shows the spectrum of the mixture containing 28% from “2 nm 3% N-doped GQD” and 62% from “2 nm 7% N-doped GQD”. This specific mixture gives rise to a fairly flat absorption spectrum in the visible region.

As for any computer simulation, the TDDFT method has errors, and these errors are usually determined by the choice of the functional and basis set. For example, for spatially extended Rydberg states, the functionals wB97XD, CAM-B3LYP, and M06-2X perform better for the complete profile of the spectra whereas B3LYP may be enough as far as the peak positions are concerned.^12,15,34^ Furthermore, to reduce the errors one may need to resort to expensive methods such as EOM-CCSD.^34^ However, a high-throughput investigation of large nanocrystals with more than 200 atoms as in this work would be prohibitively expensive, if not impossible, both in computational resources as well as human workforce. While these considerations justify the practical and necessary choice of TDDFT method at the B3LYP/6-31G(d) level for the GQDs up to 2 nm here, the results presented should not be seen as a precision study; instead, the general systematics and trends in the results should be the main lesson to be taken here.

## Conclusions and outlook

We presented a TDDFT study of 63 different GQDs with systematically varying size, dopant type, and dopant percentage. The results suggested visible trends in the peak properties as well as the general profile in the absorption spectrum. The TDDFT calculations here shed light on the systematics in the absorption properties of the nanometer-sized graphene nanocrystals investigated in this work. A desired spectrum can be obtained by mixing different GQDs with appropriate proportions, and the spectra calculated in this work can be utilized to this end. This study may be extended to codoping cases, different solvents, surface functionals, and other structural modifications of GQDs.

## Supporting Information Available

UV–vis absorption spectra of 63 graphene quantum dots (PDF)
