# Supplementary material for: Doped Graphene Quantum Dots UV–vis Absorption Spectrum: A High-Throughput TDDFT Study
Source: ACS Omega. 2023 Jan 5;8(2):2112–8. doi: 10.1021/acsomega.2c06091 (PMC9850463; doi:10.1021/acsomega.2c06091)
Supplement: Supplementary file 1 — ao2c06091_si_001.pdf [file ao2c06091_si_001.pdf]

Supporting Information: Doped Graphene Quantum Dots UV-Vis Absorption Spectrum: A high-throughput TDDFT study

Şener Özönder
Institute for Data Science & Artificial Intelligence, Boğaziçi University, Istanbul, Türkiye[*]

Caner Ünlü
Department of Chemistry, Istanbul Technical University, Istanbul, Türkiye

Cihat Güleryüz
Department of Physics, Marmara University, Istanbul, Türkiye and
Department of Opticianry, Altınbaş University, Istanbul, Türkiye

Levent Trabzon
Department of Mechanical Engineering, Istanbul Technical University, Istanbul, Türkiye

[*] Corresponding author: sener.ozonder@boun.edu.tr

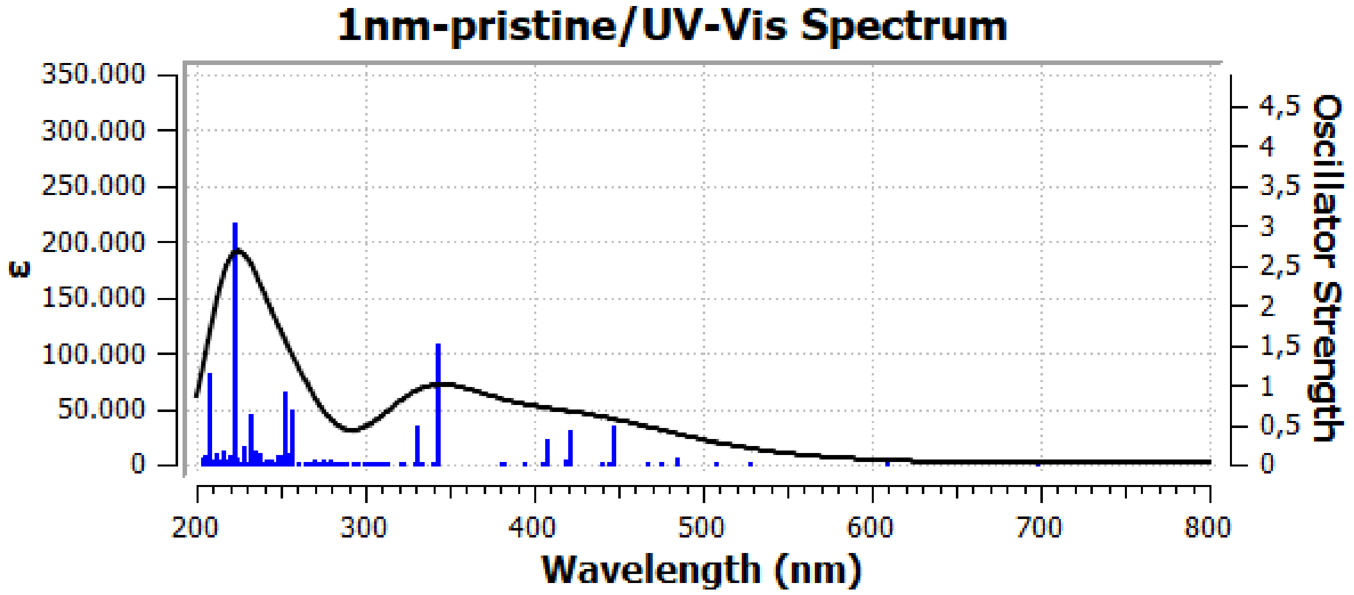

Figure S1. UV-Vis spectrum of 1 nm pristine graphene.

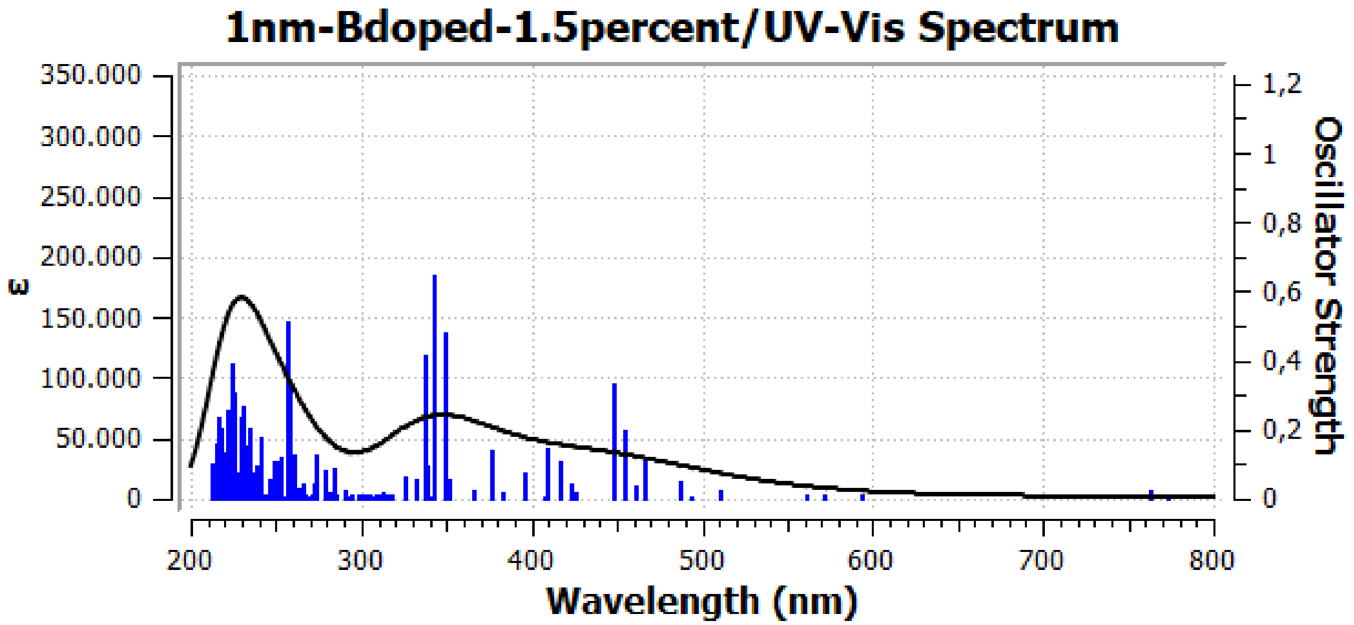

Figure S2. UV-Vis spectrum of 1 nm 1.5% B-doped graphene.

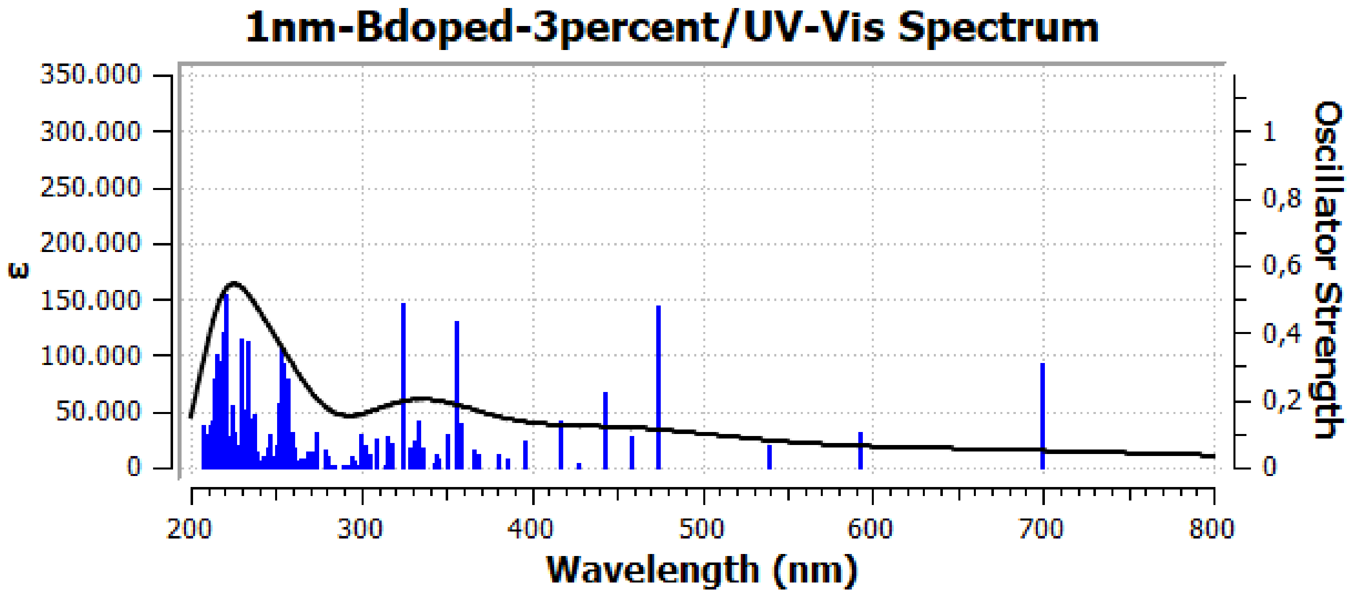

Figure S3. UV-Vis spectrum of 1 nm 3% B-doped graphene.

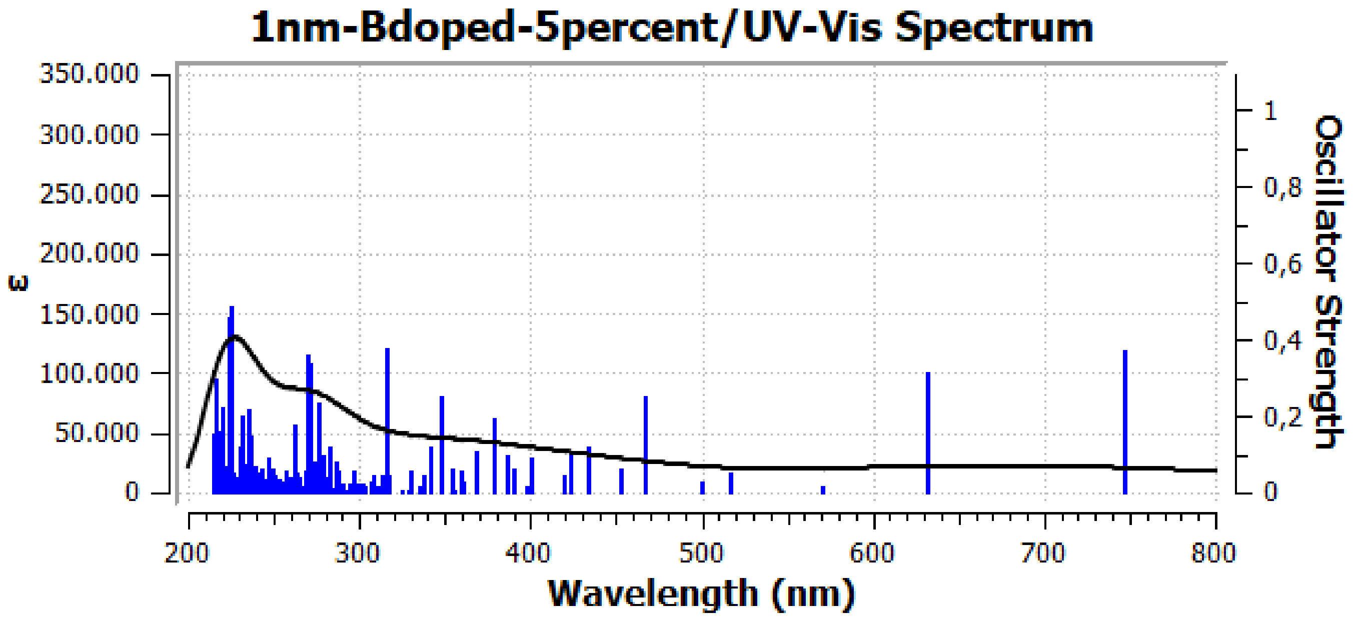

Figure S4. UV-Vis spectrum of 1 nm 5% B-doped graphene.

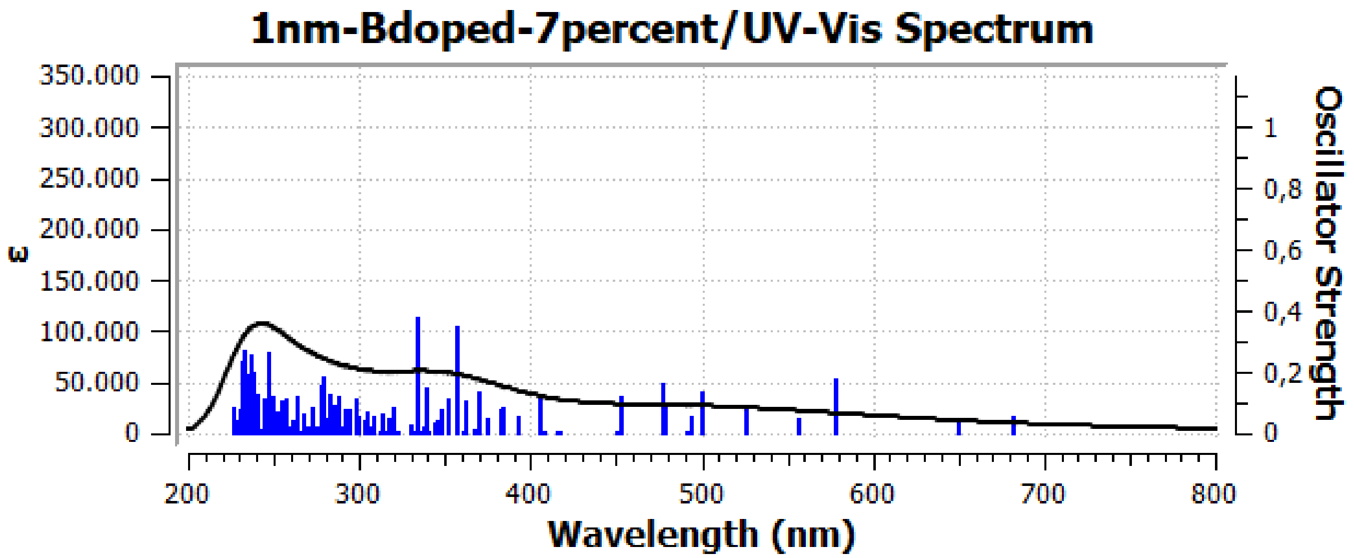

Figure S5. UV-Vis spectrum of 1 nm 7% B-doped graphene.

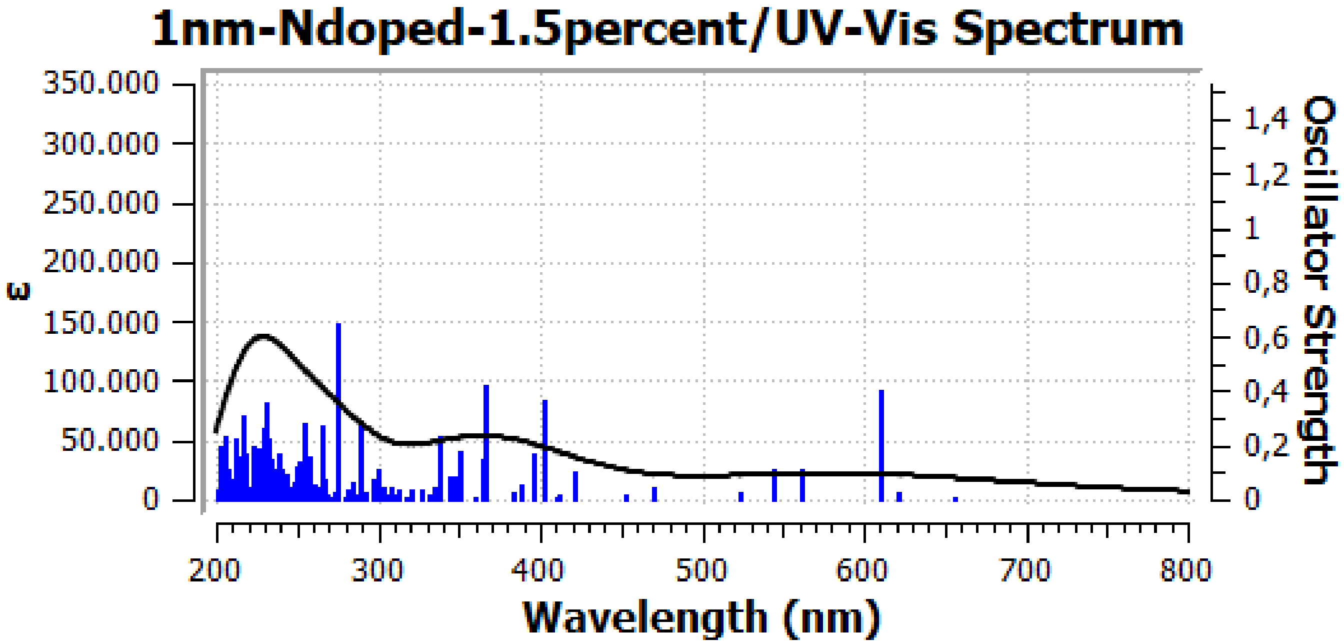

Figure S6. UV-Vis spectrum of 1 nm 1.5% N-doped graphene.

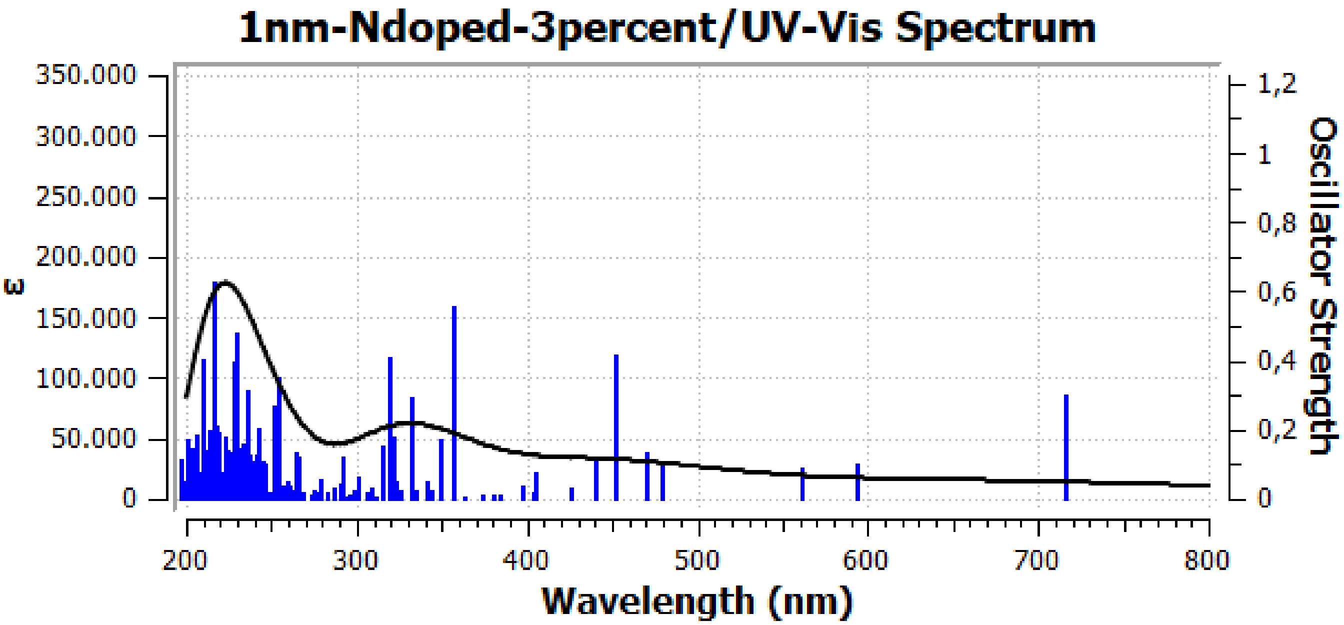

Figure S7. UV-Vis spectrum of 1 nm 3% N-doped graphene.

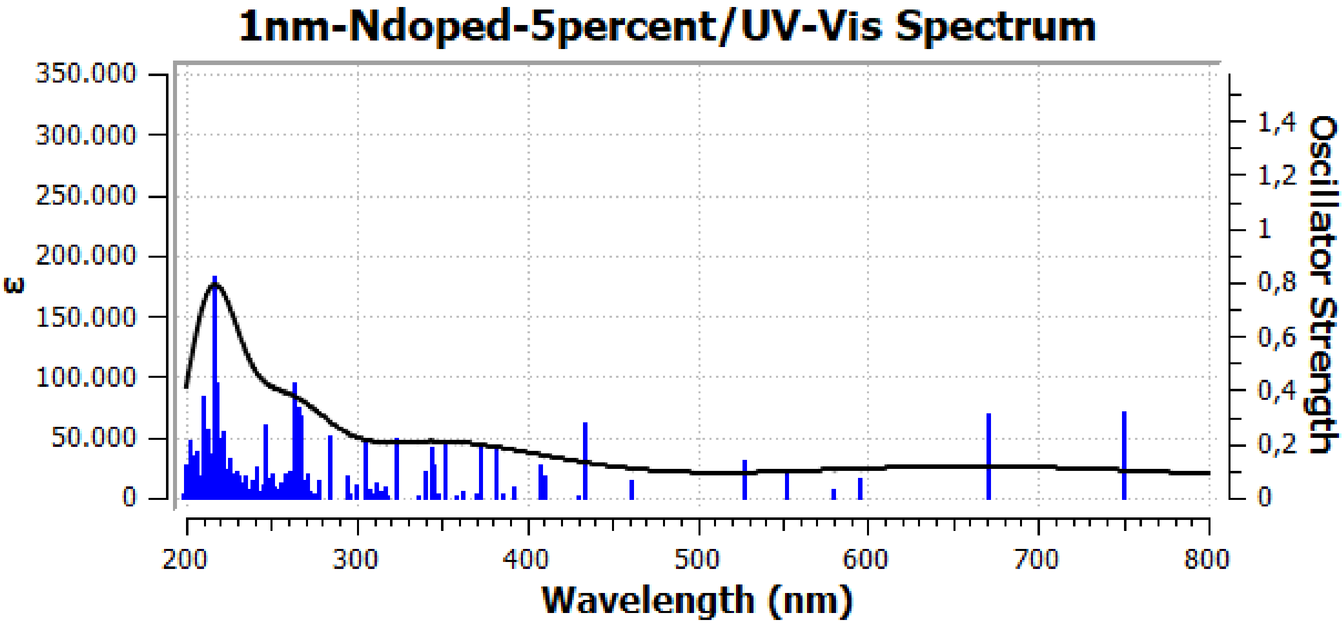

Figure S8. UV-Vis spectrum of 1 nm 5% N-doped graphene.

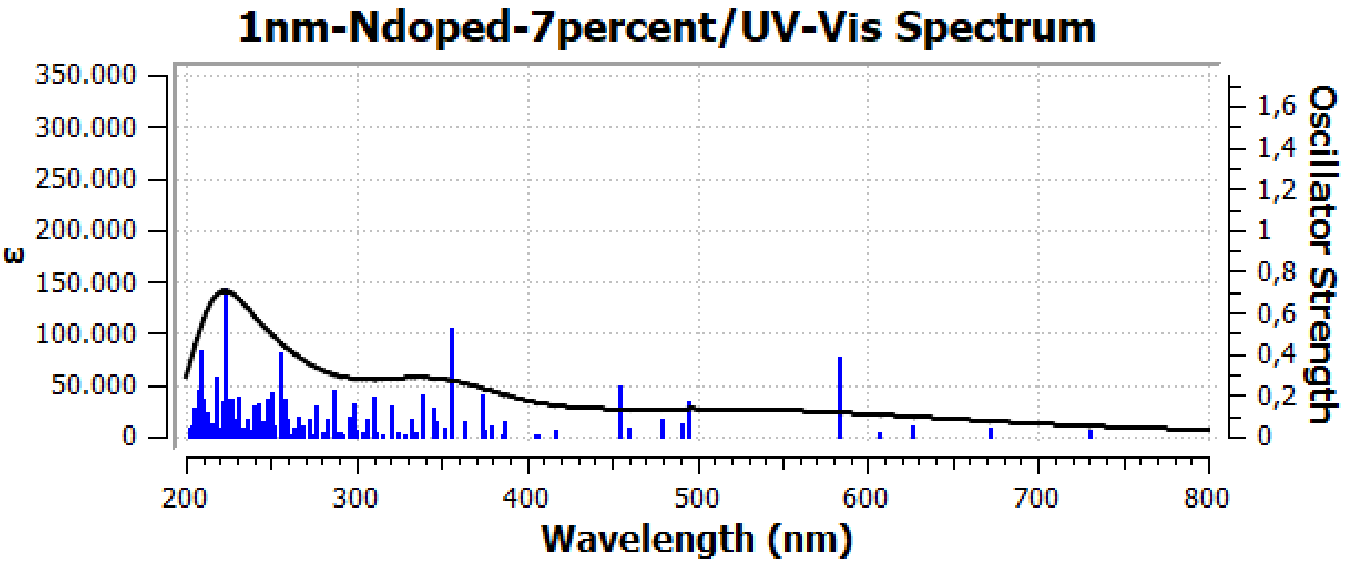

Figure S9. UV-Vis spectrum of 1 nm 7% N-doped graphene.

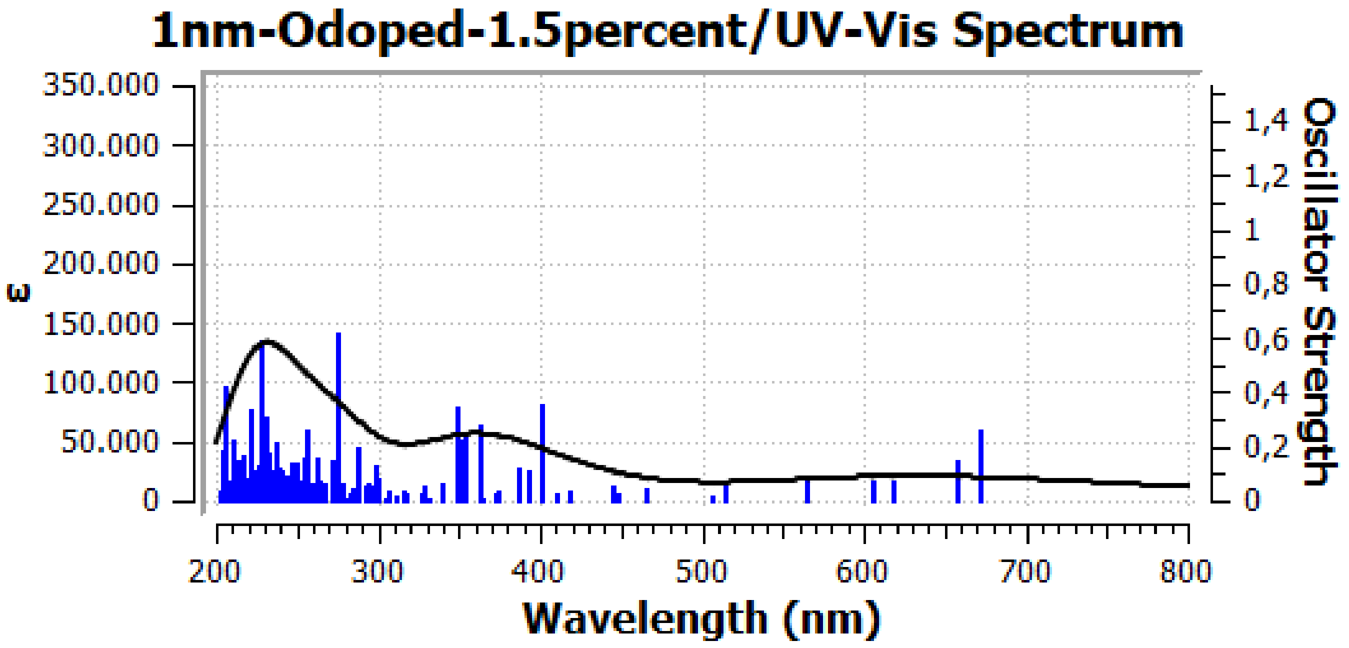

Figure S10. UV-Vis spectrum of 1 nm 1.5% O-doped graphene.

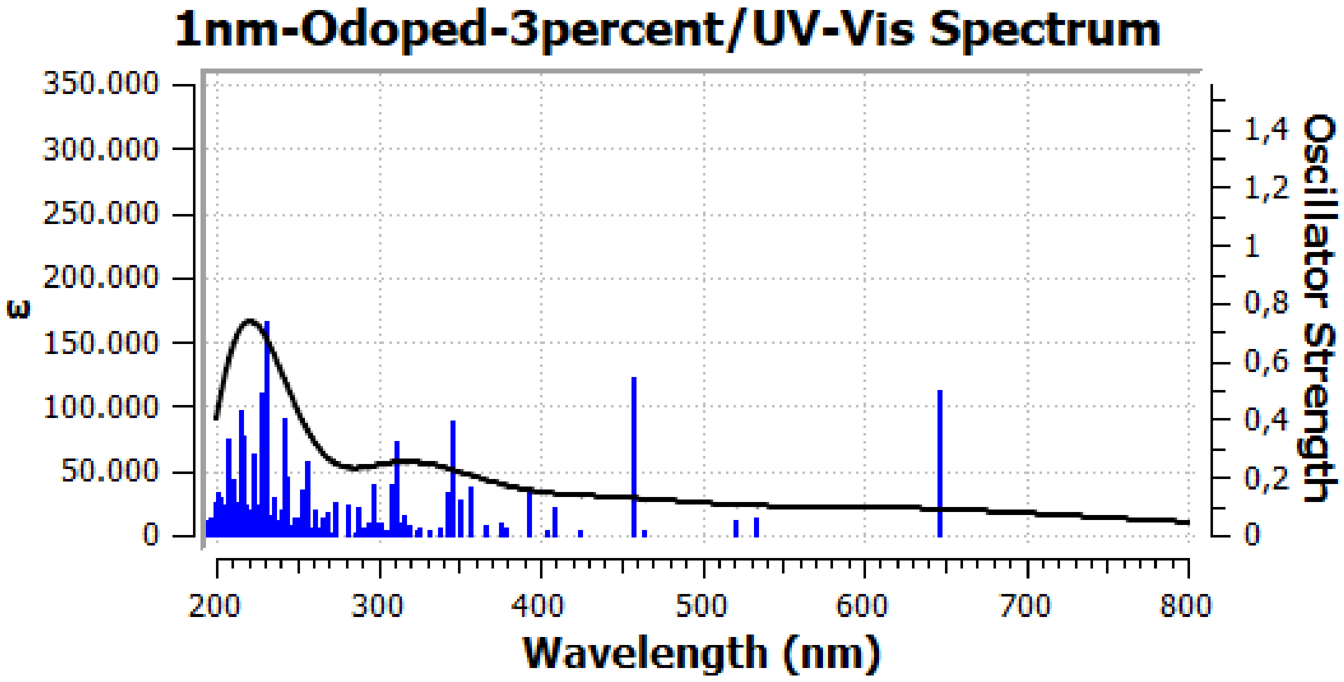

Figure S11. UV-Vis spectrum of 1 nm 3% O-doped graphene.

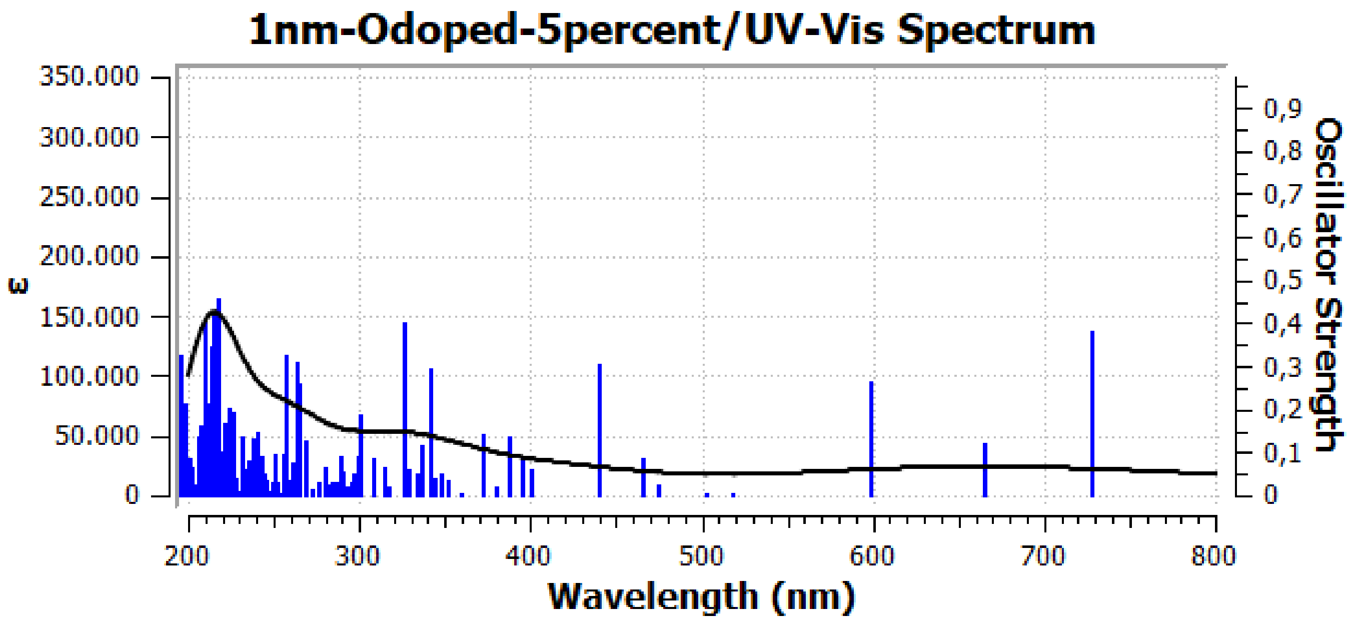

Figure S12. UV-Vis spectrum of 1 nm 5% O-doped graphene.

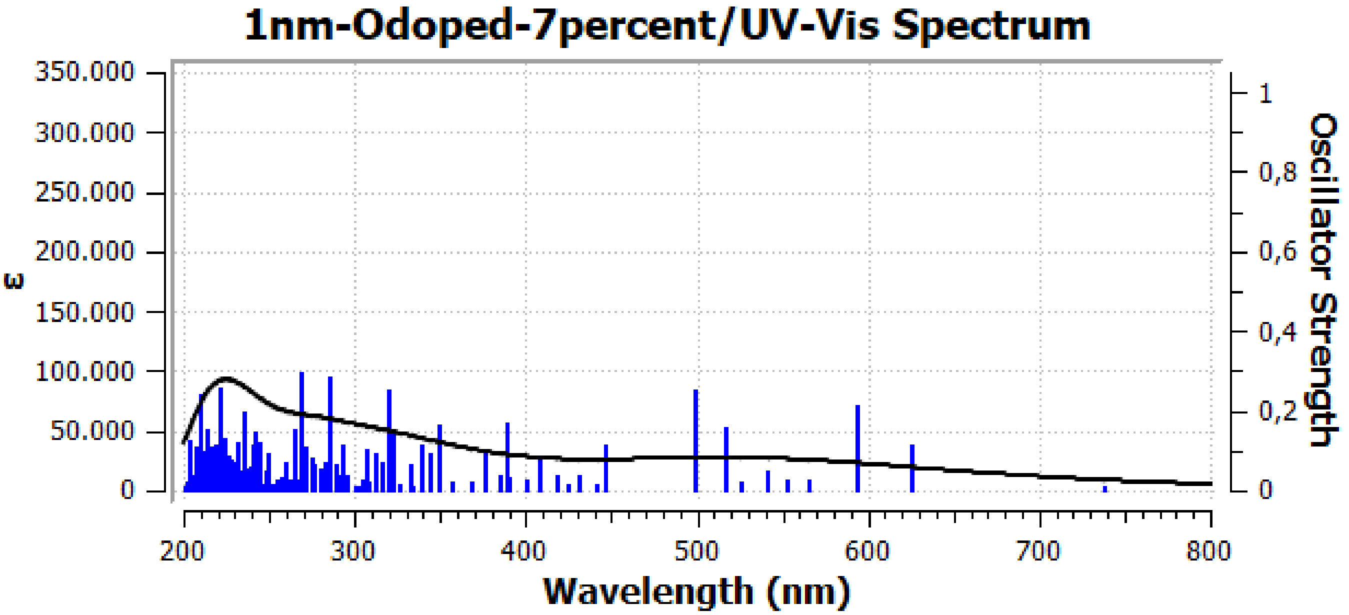

Figure S13. UV-Vis spectrum of 1 nm 7% O-doped graphene.

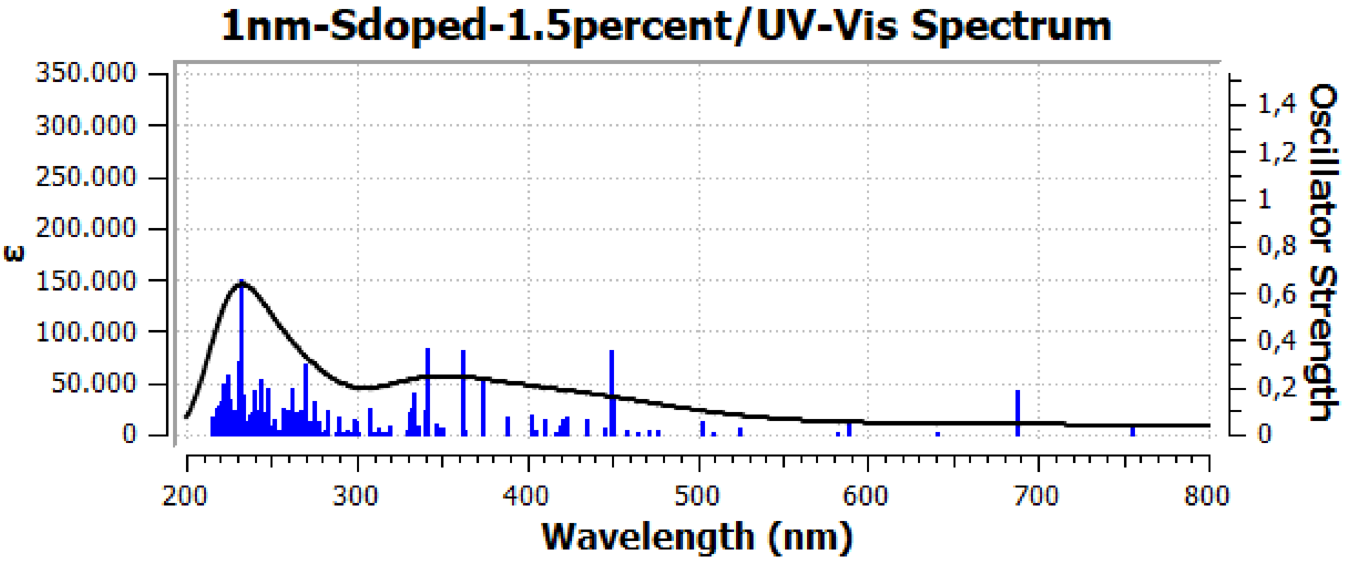

Figure S14. UV-Vis spectrum of 1 nm 1.5% S-doped graphene.

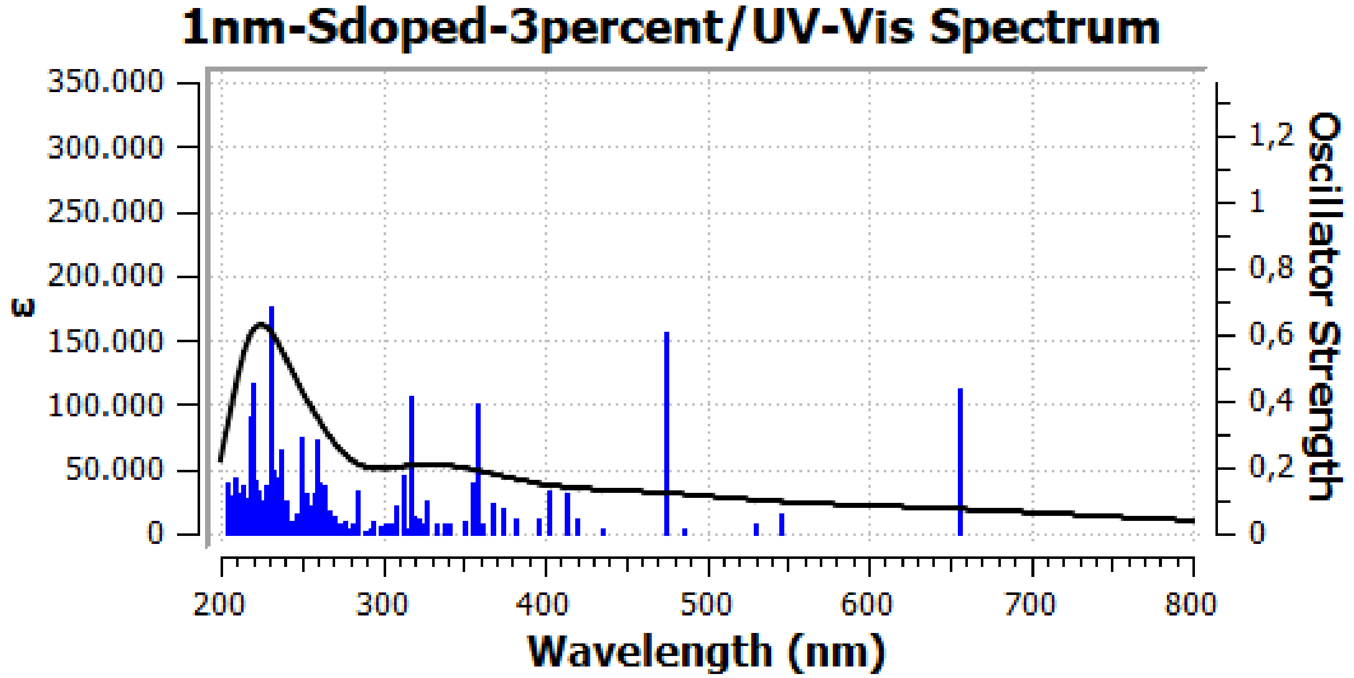

Figure S15. UV-Vis spectrum of 1 nm 3% S-doped graphene.

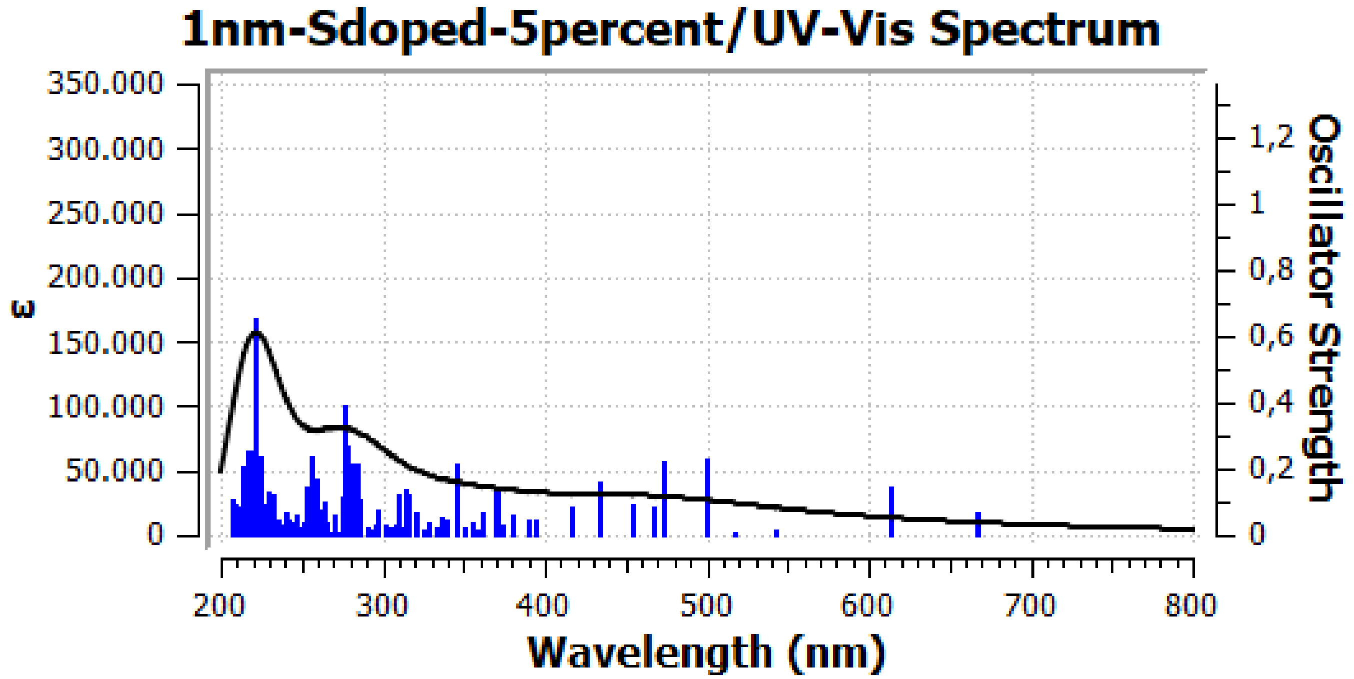

Figure S16. UV-Vis spectrum of 1 nm 5% S-doped graphene.

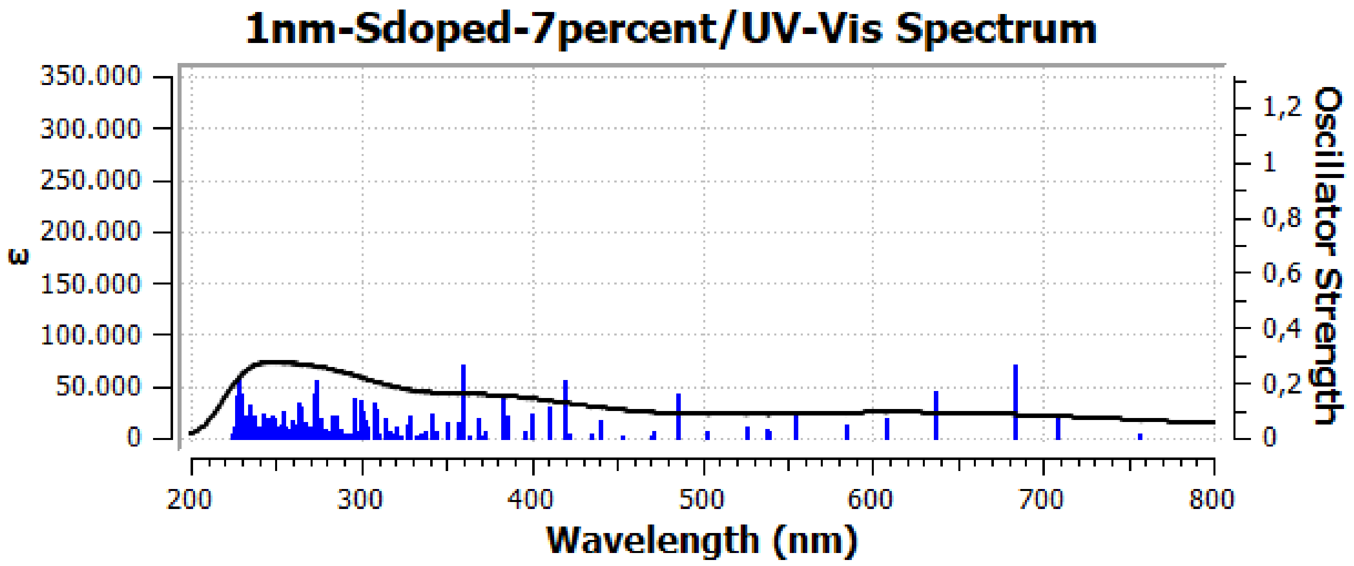

Figure S17. UV-Vis spectrum of 1 nm 7% S-doped graphene.

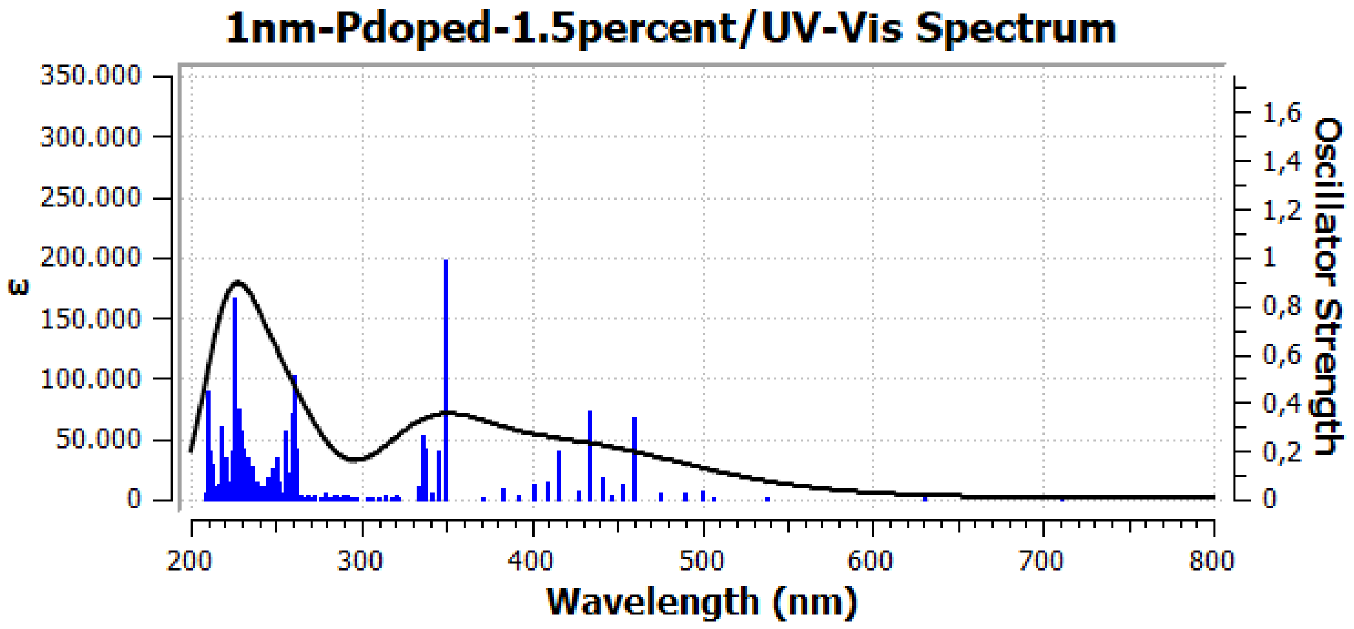

Figure S18. UV-Vis spectrum of 1 nm 1.5% P-doped graphene.

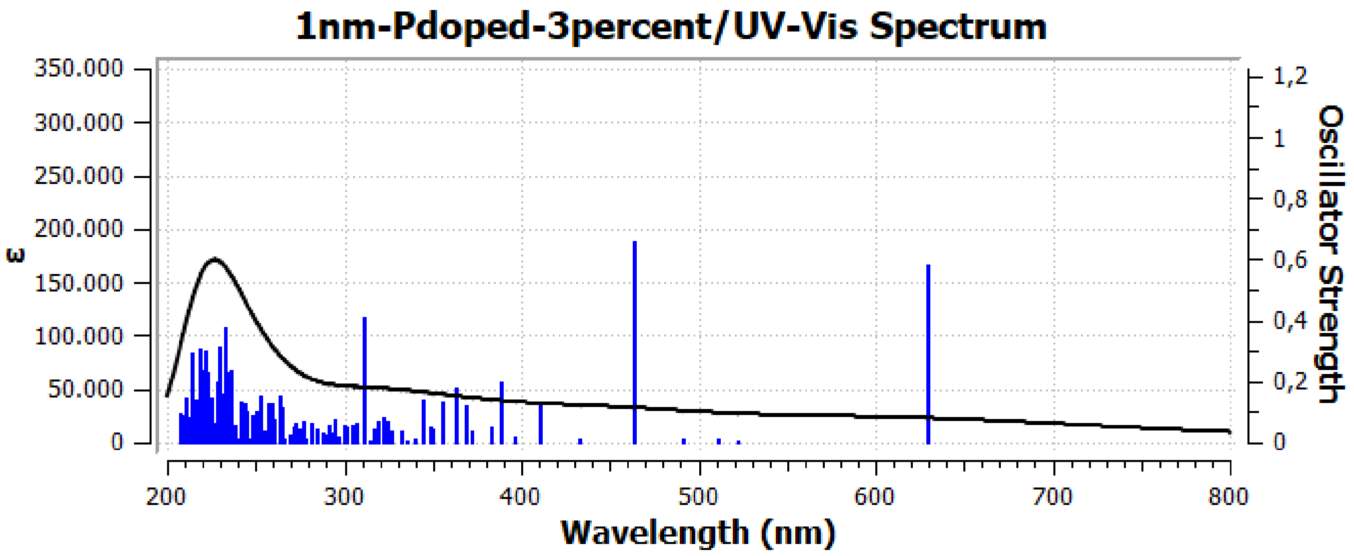

Figure S19. UV-Vis spectrum of 1 nm 3% P-doped graphene.

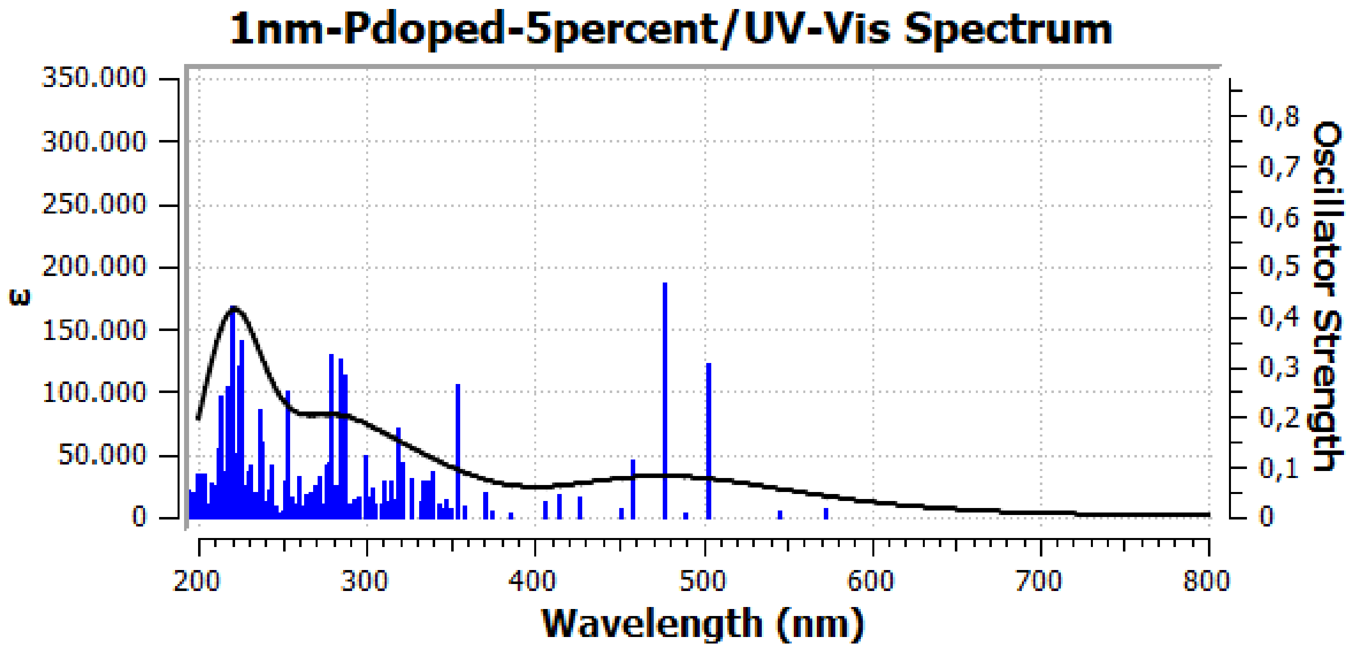

Figure S20. UV-Vis spectrum of 1 nm 5% P-doped graphene.

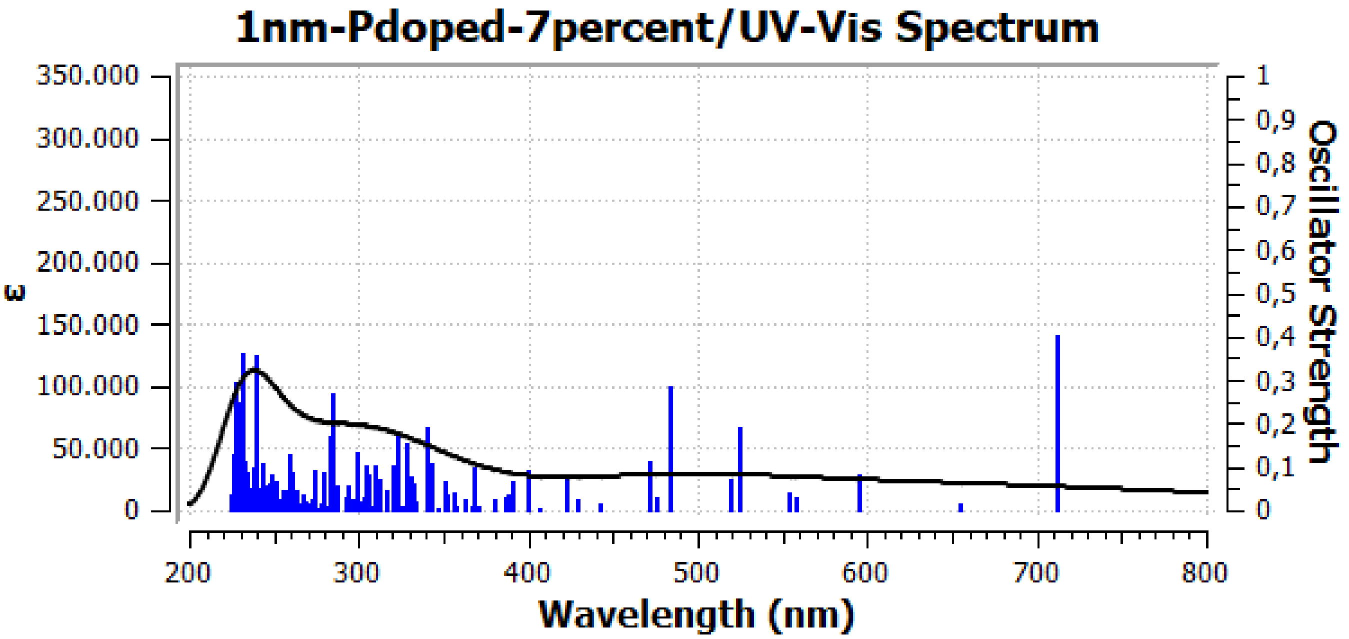

Figure S21. UV-Vis spectrum of 1 nm 7% P-doped graphene.

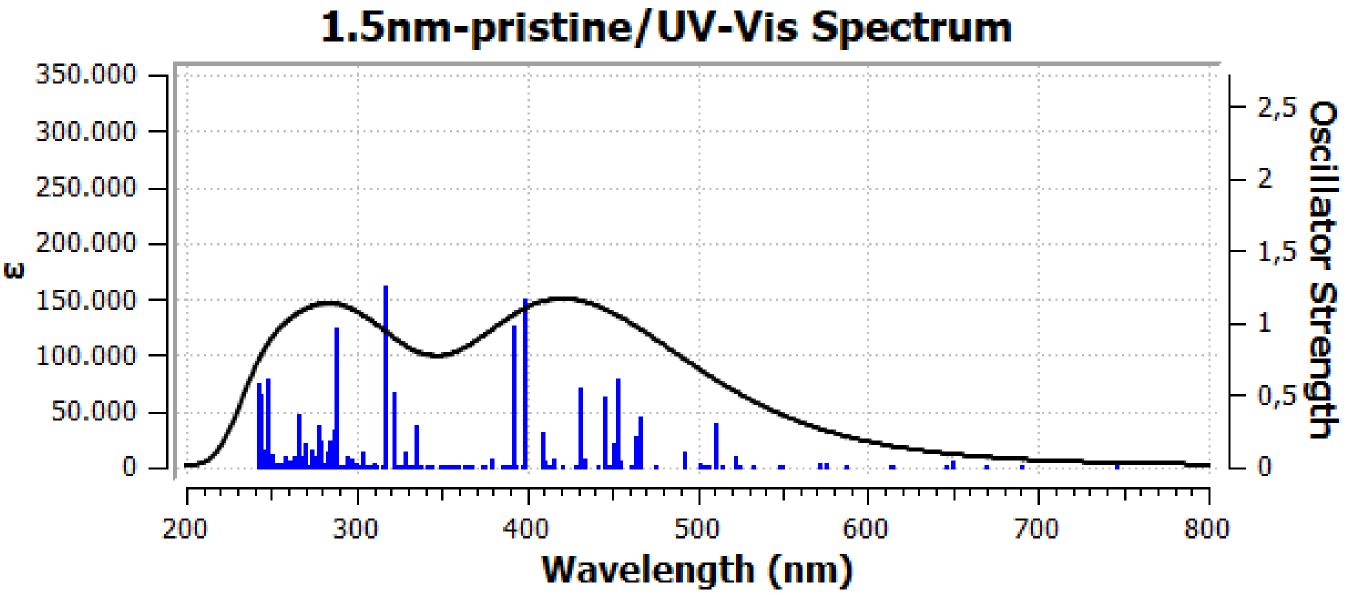

Figure S22. UV-Vis spectrum of 1.5 nm pristine graphene.

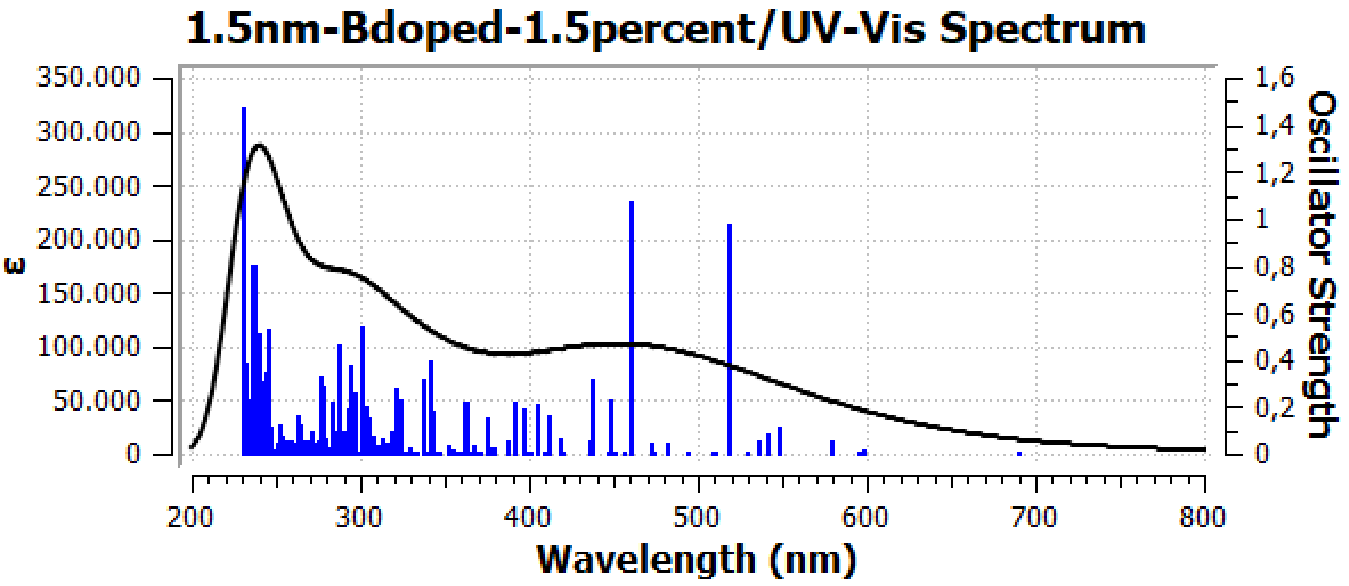

Figure S23. UV-Vis spectrum of 1.5 nm 1.5% B-doped graphene.

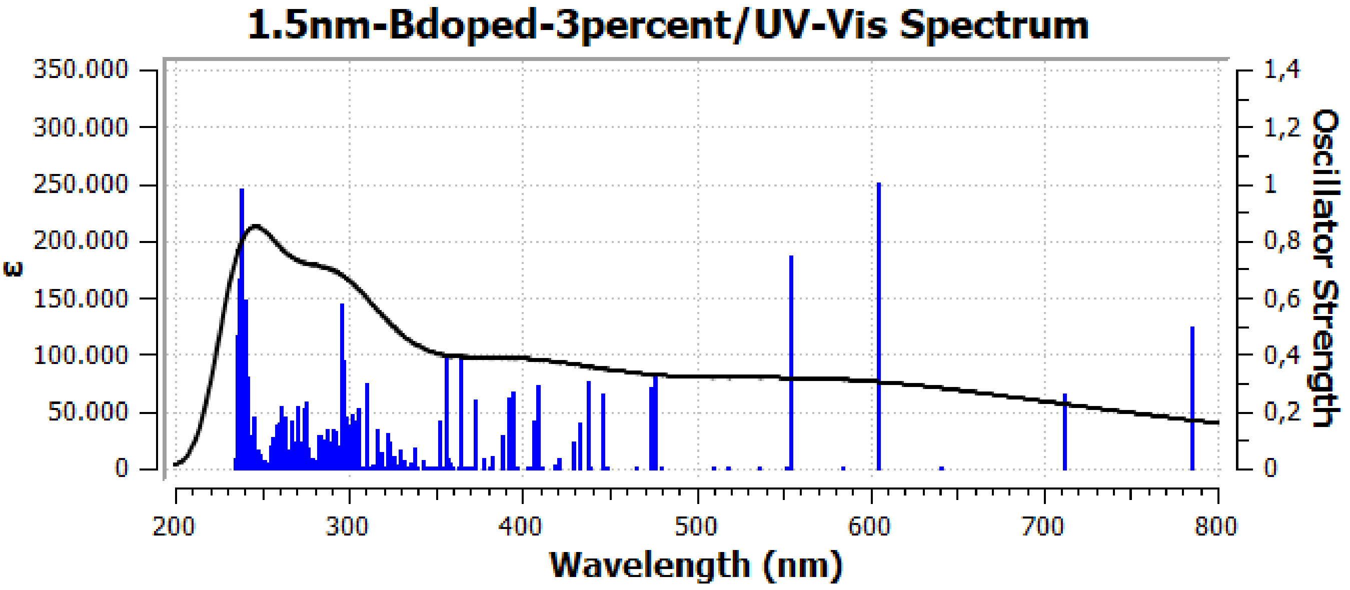

Figure S24. UV-Vis spectrum of 1.5 nm 3% B-doped graphene.

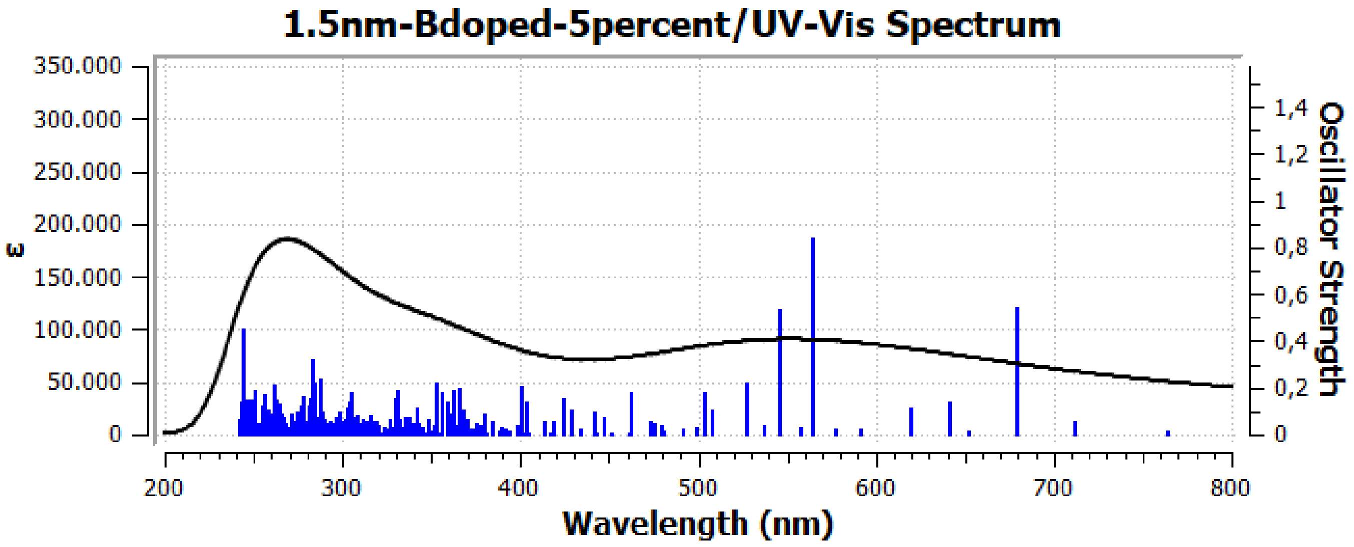

Figure S25. UV-Vis spectrum of 1.5 nm 5% B-doped graphene.

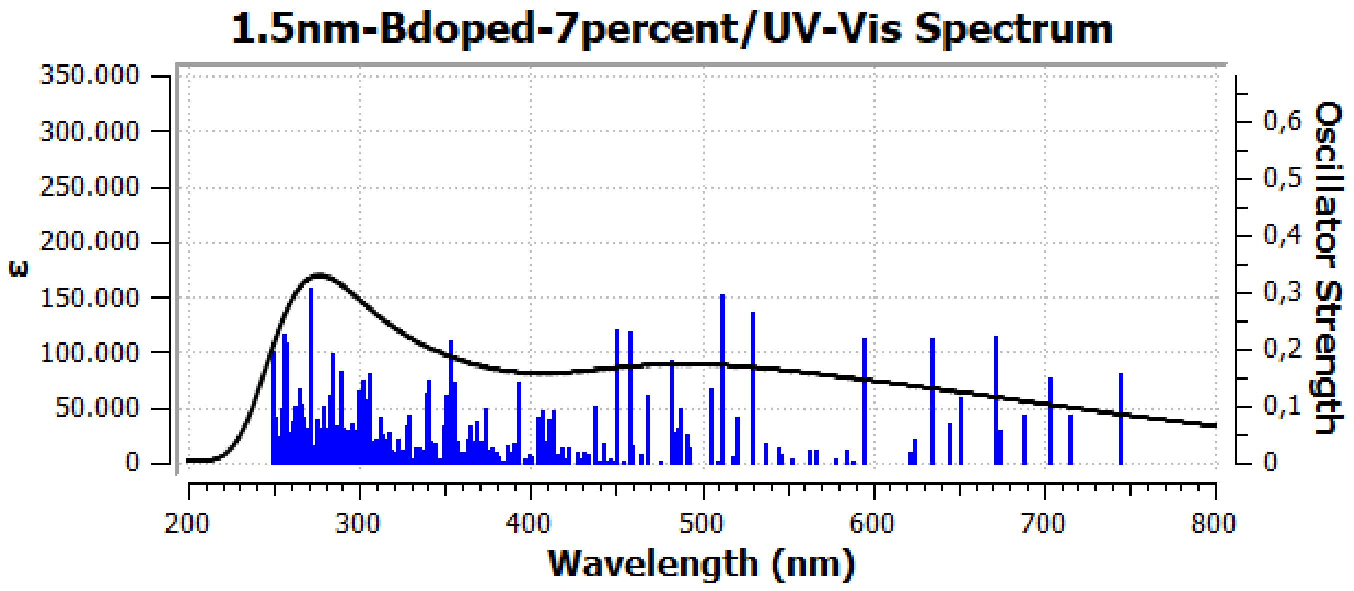

Figure S26. UV-Vis spectrum of 1.5 nm 7% B-doped graphene.

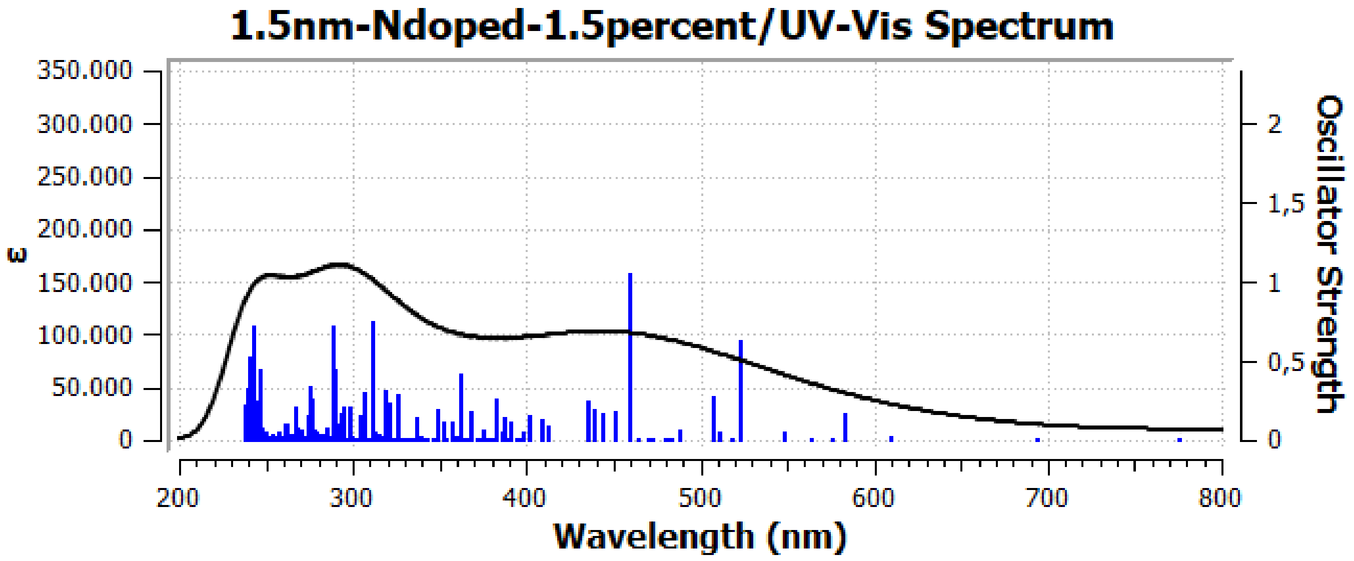

Figure S27. UV-Vis spectrum of 1.5 nm 1.5% N-doped graphene.

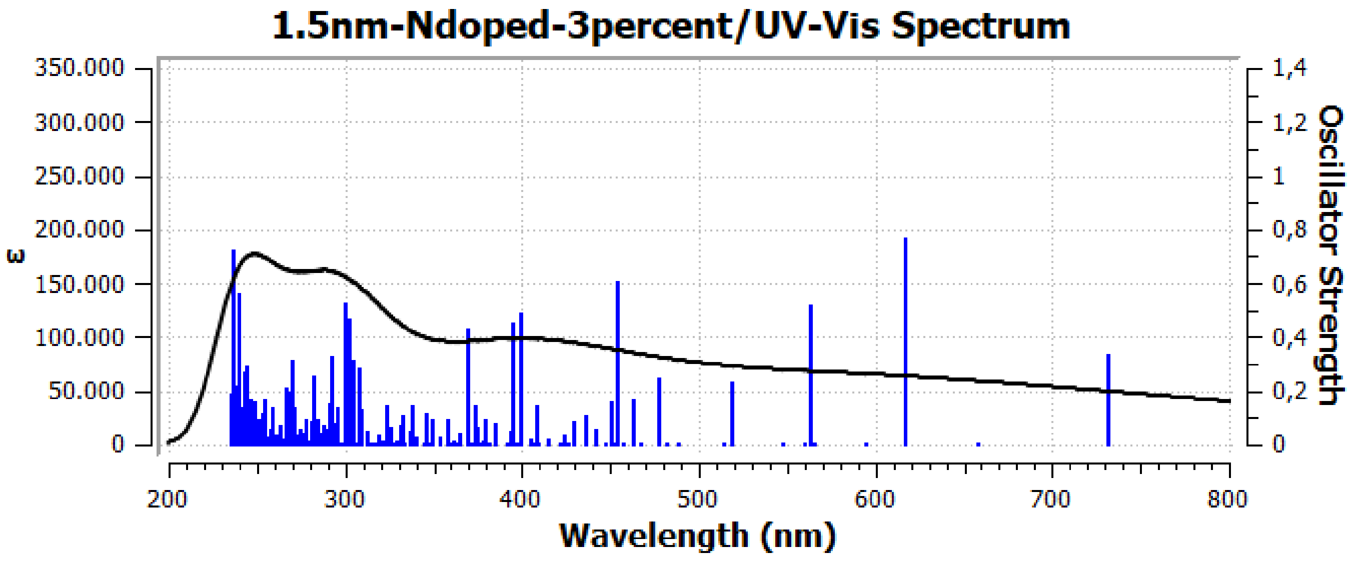

Figure S28. UV-Vis spectrum of 1.5 nm 3% N-doped graphene.

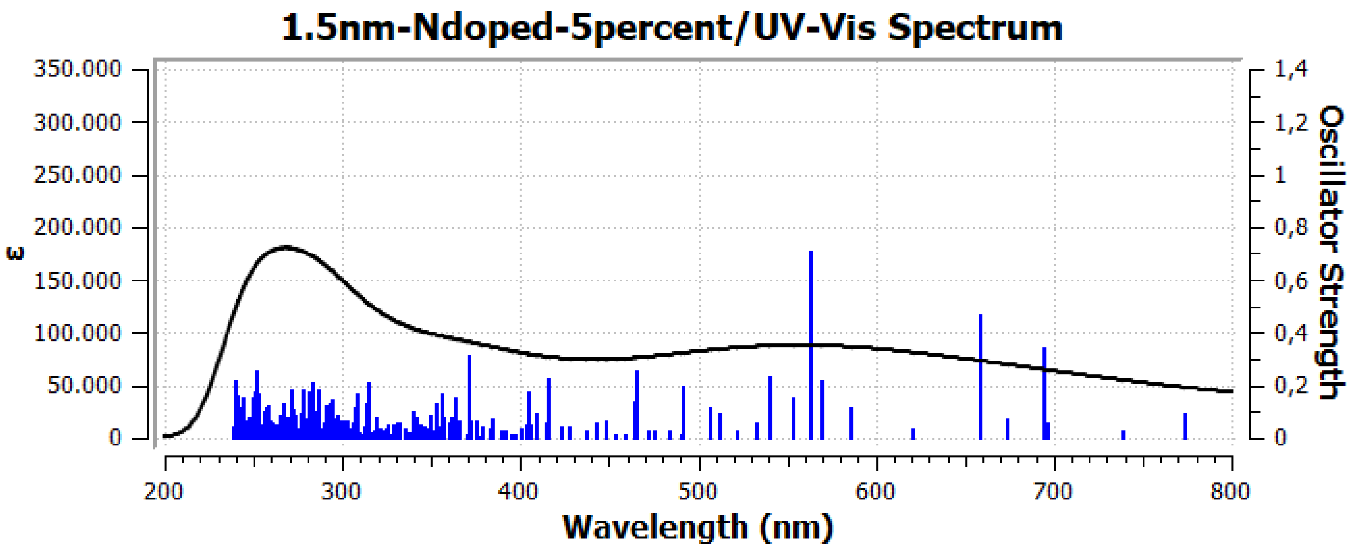

Figure S29. UV-Vis spectrum of 1.5 nm 5% N-doped graphene.

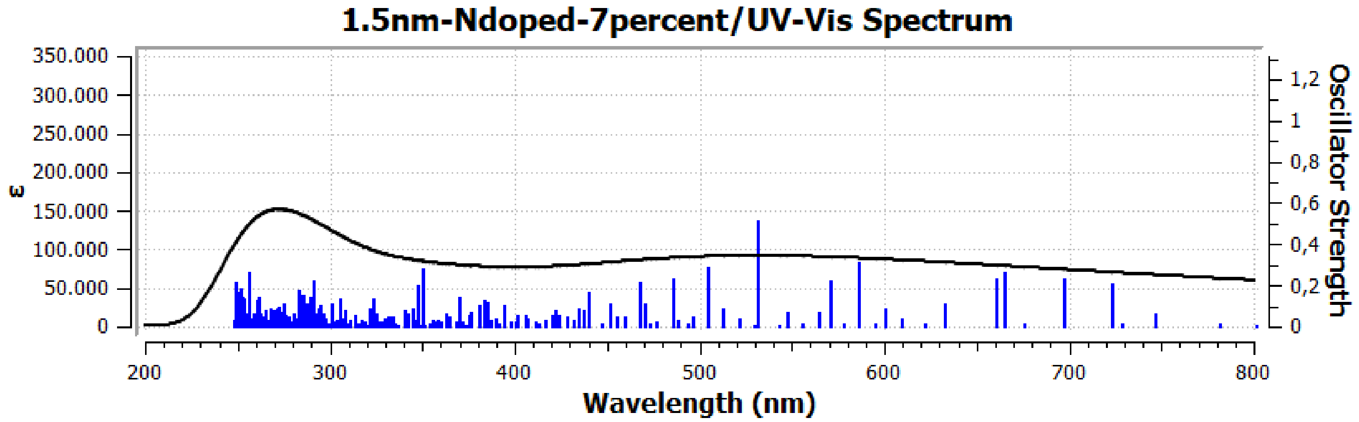

Figure S30. UV-Vis spectrum of 1.5 nm 7% N-doped graphene.

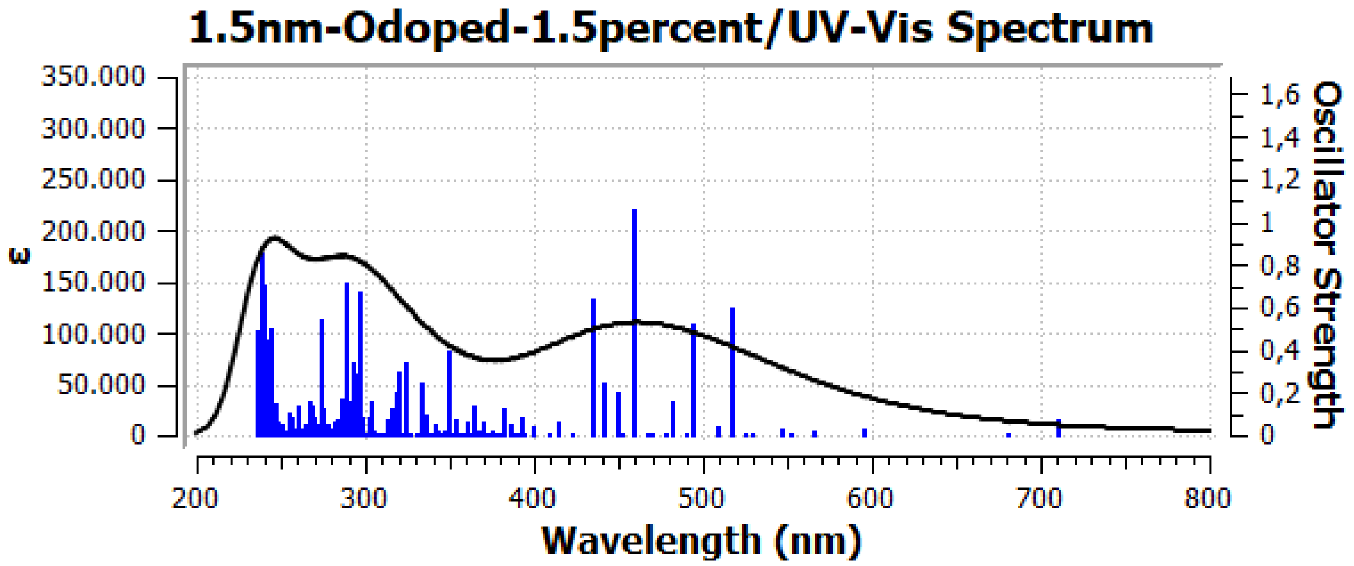

Figure S31. UV-Vis spectrum of 1.5 nm 1.5% O-doped graphene.

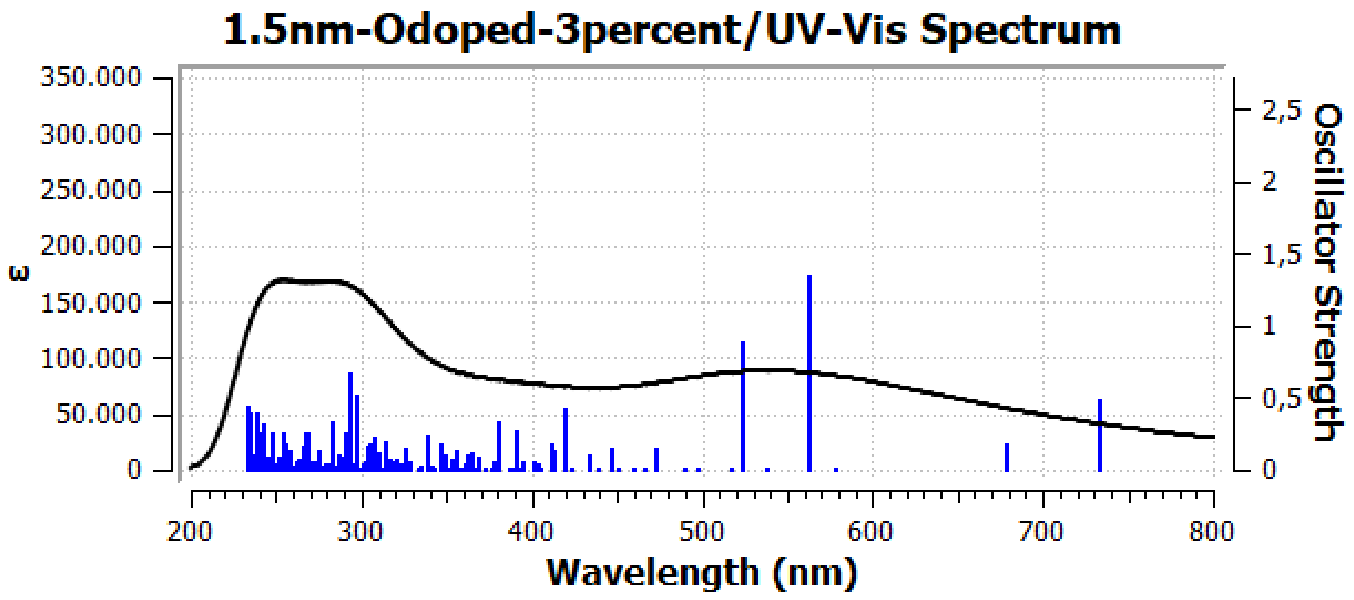

Figure S32. UV-Vis spectrum of 1.5 nm 3% O-doped graphene.

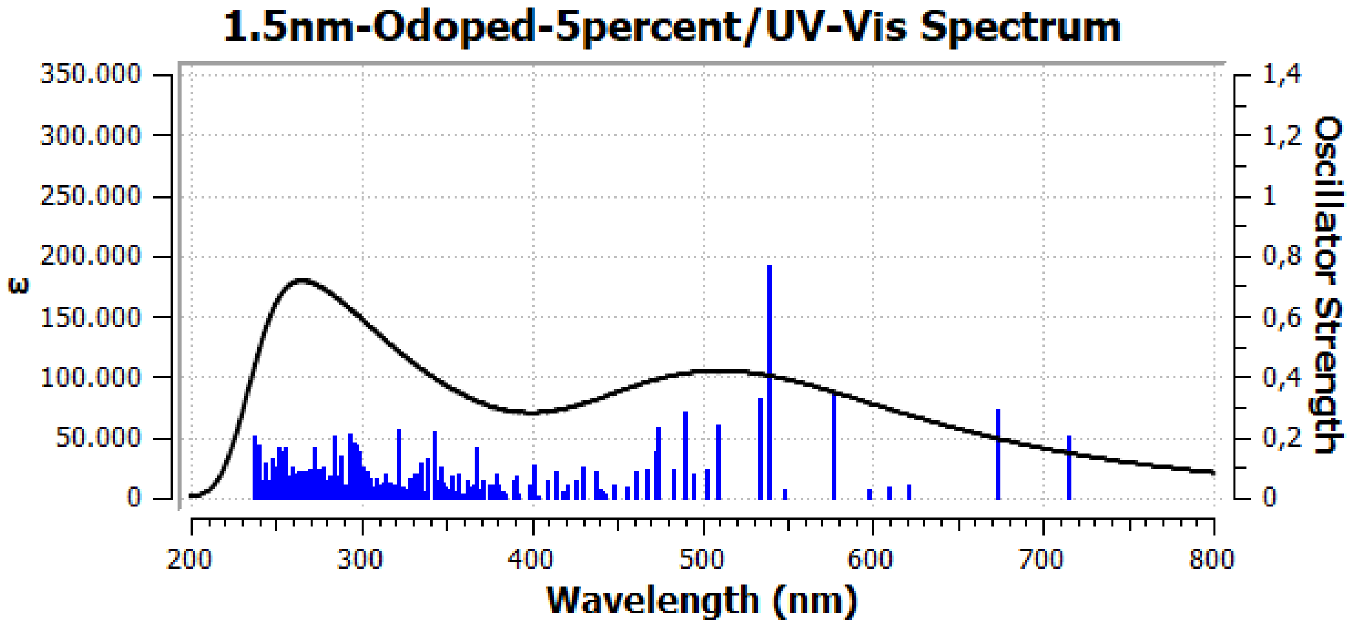

Figure S33. UV-Vis spectrum of 1.5 nm 5% O-doped graphene.

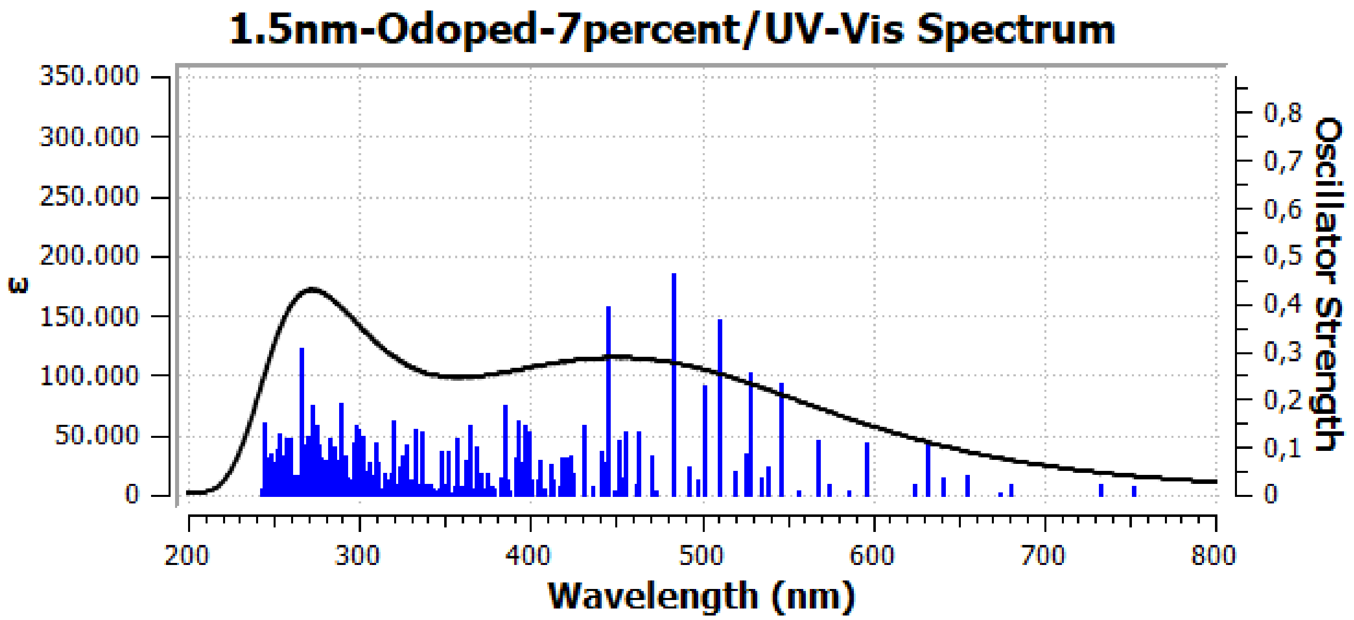

Figure S34. UV-Vis spectrum of 1.5 nm 7% O-doped graphene.

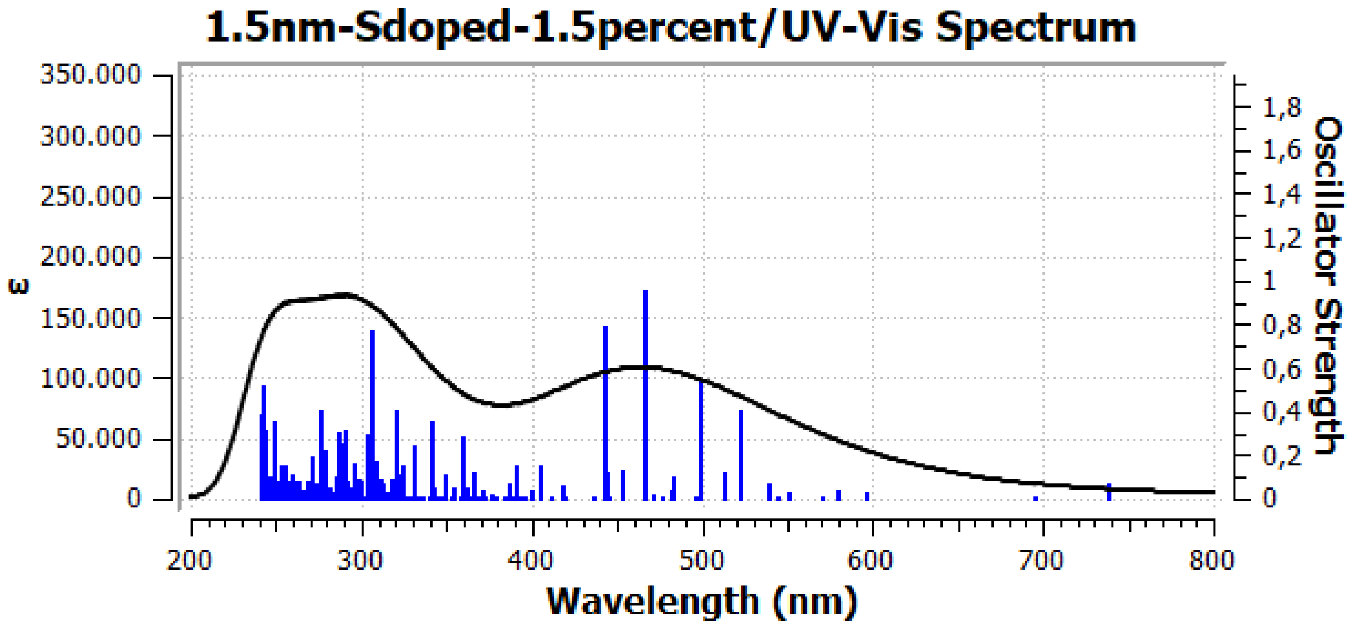

Figure S35. UV-Vis spectrum of 1.5 nm 1.5% S-doped graphene.

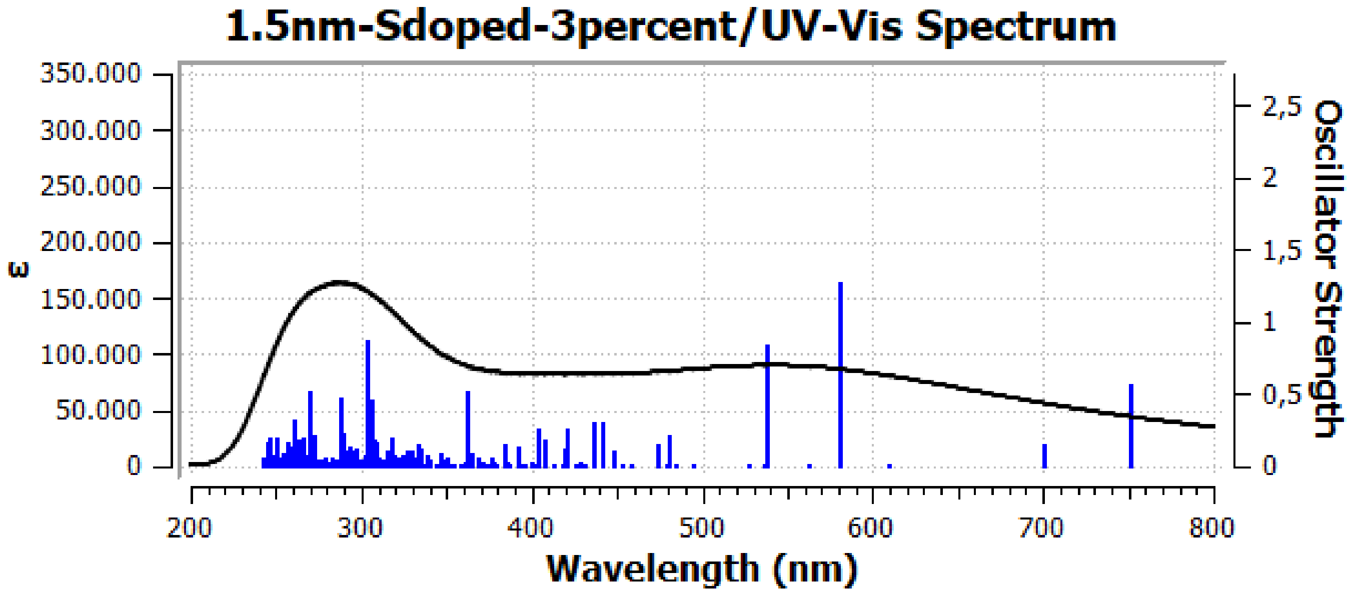

Figure S36. UV-Vis spectrum of 1.5 nm 3% S-doped graphene.

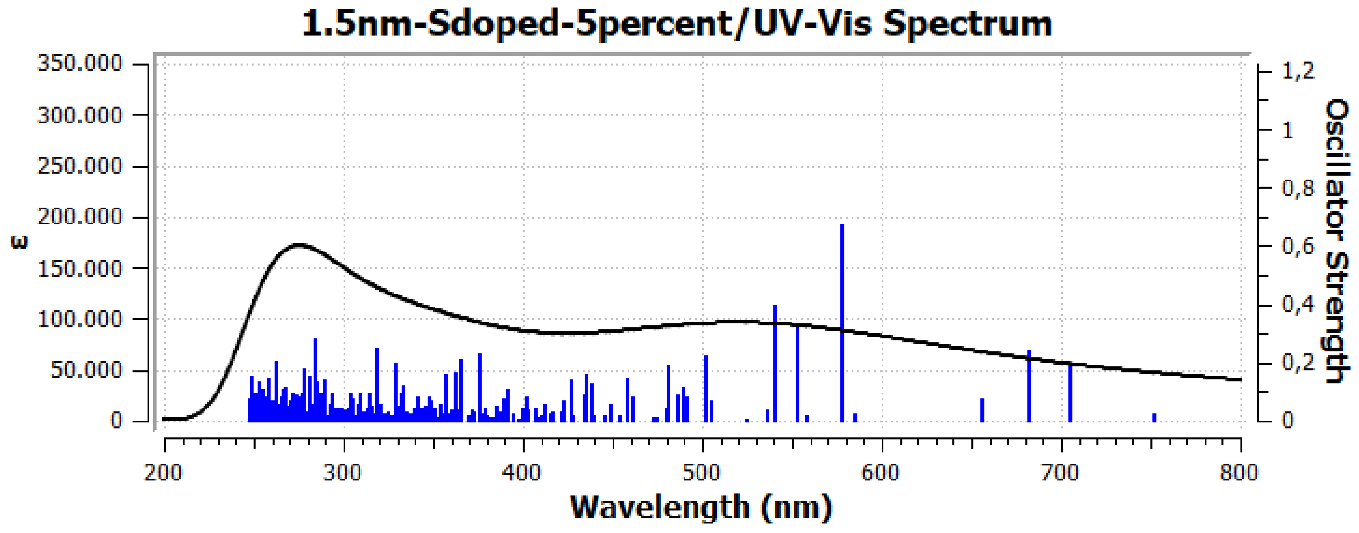

Figure S37. UV-Vis spectrum of 1.5 nm 5% S-doped graphene.

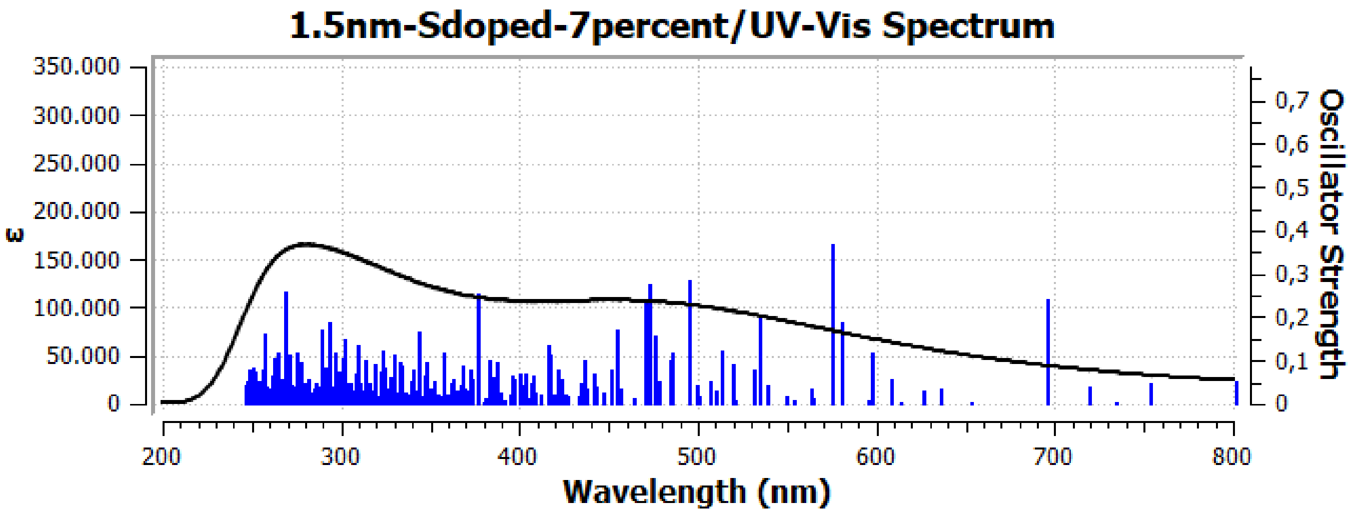

Figure S38. UV-Vis spectrum of 1.5 nm 7% S-doped graphene.

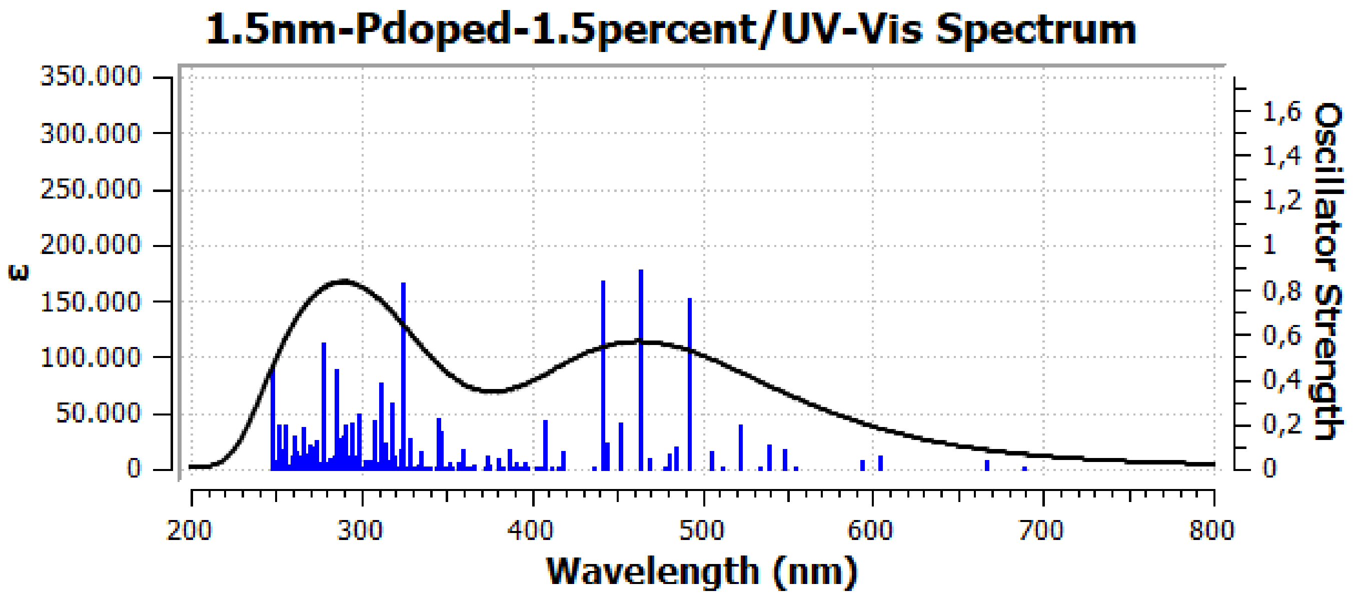

Figure S39. UV-Vis spectrum of 1.5 nm 1.5% P-doped graphene.

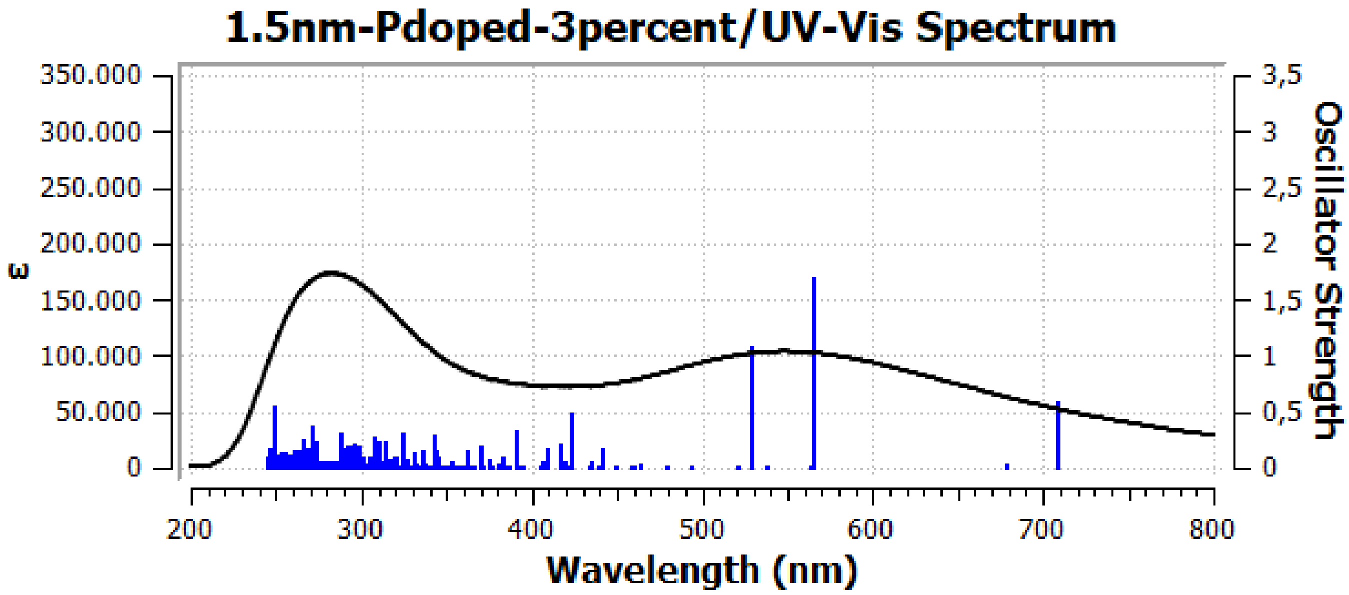

Figure S40. UV-Vis spectrum of 1.5 nm 3% P-doped graphene.

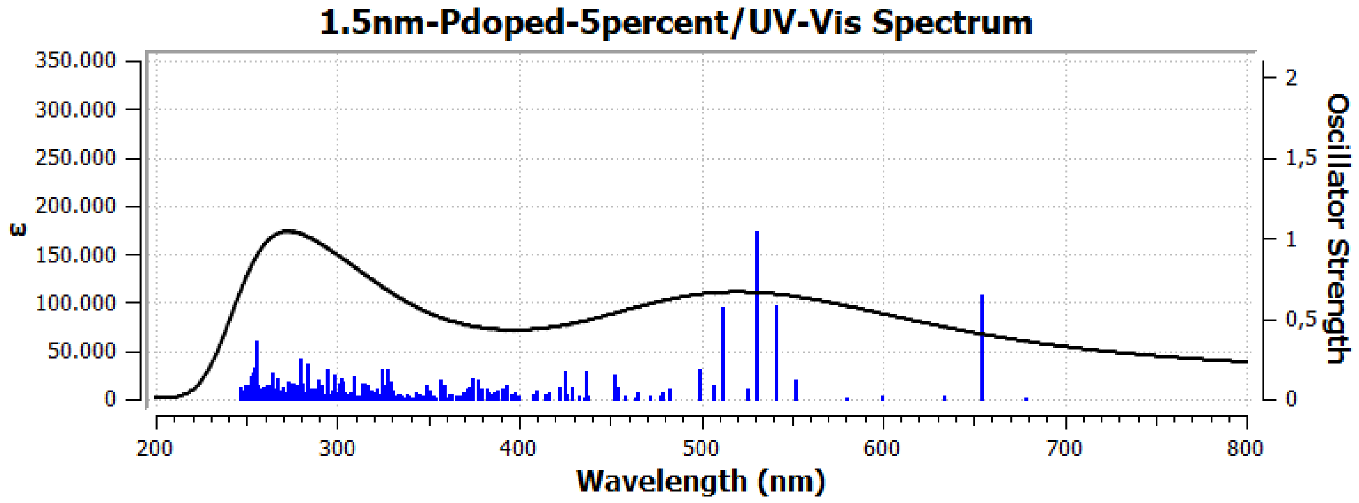

Figure S41. UV-Vis spectrum of 1.5 nm 5% P-doped graphene.

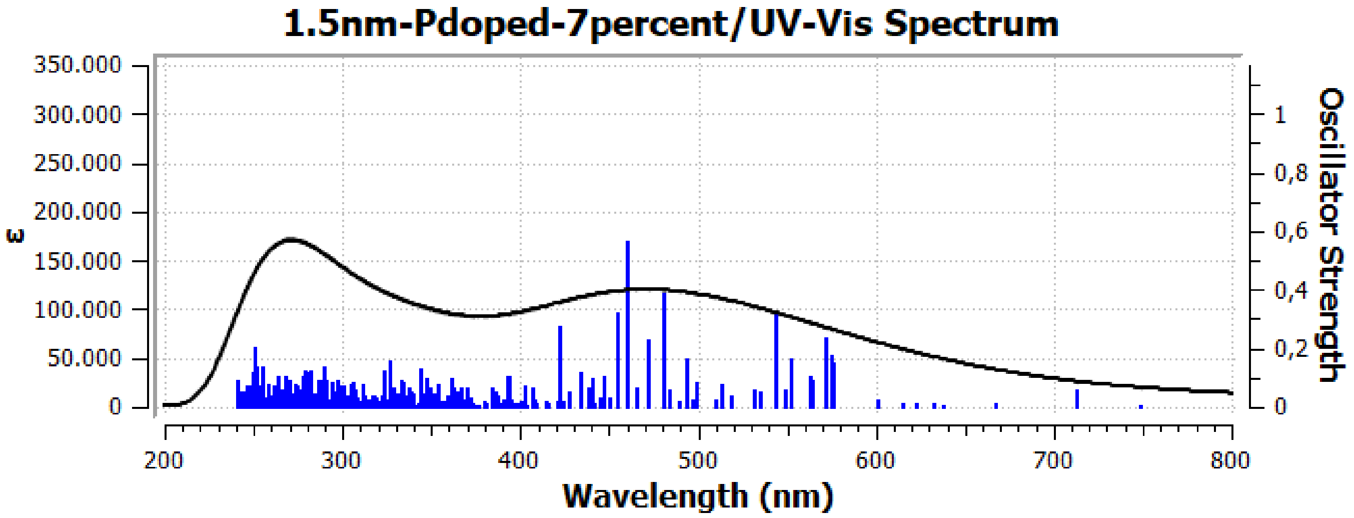

Figure S42. UV-Vis spectrum of 1.5 nm 7% P-doped graphene.

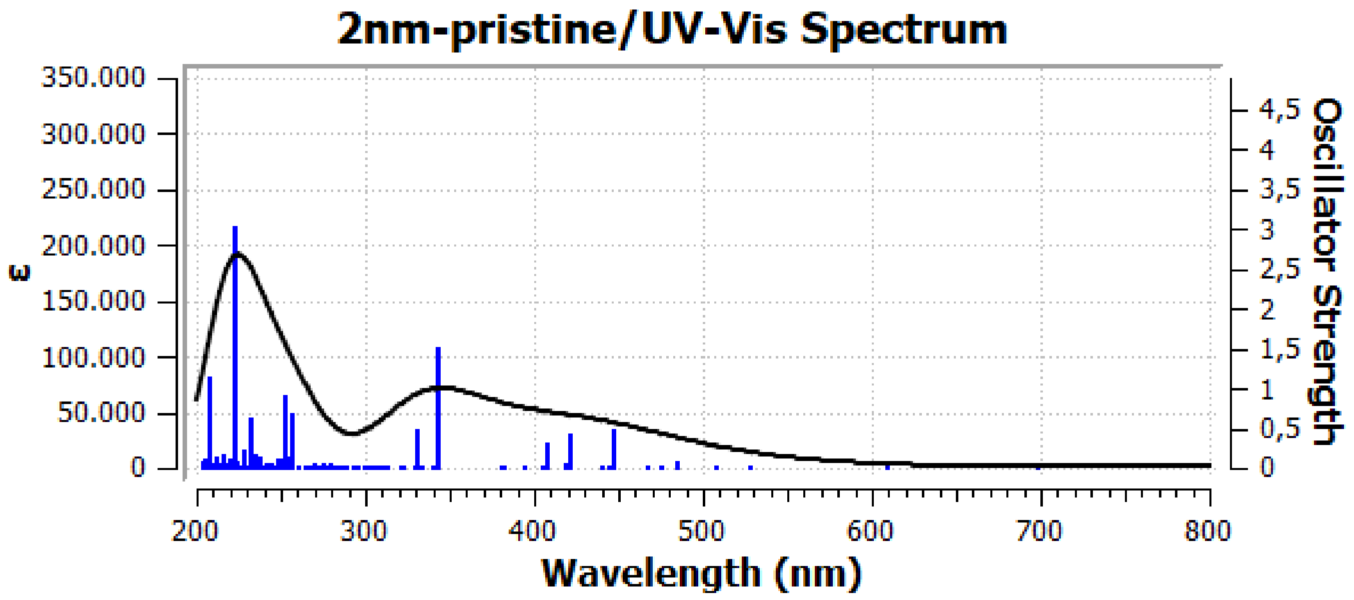

Figure S43. UV-Vis spectrum of 2 nm pristine graphene.

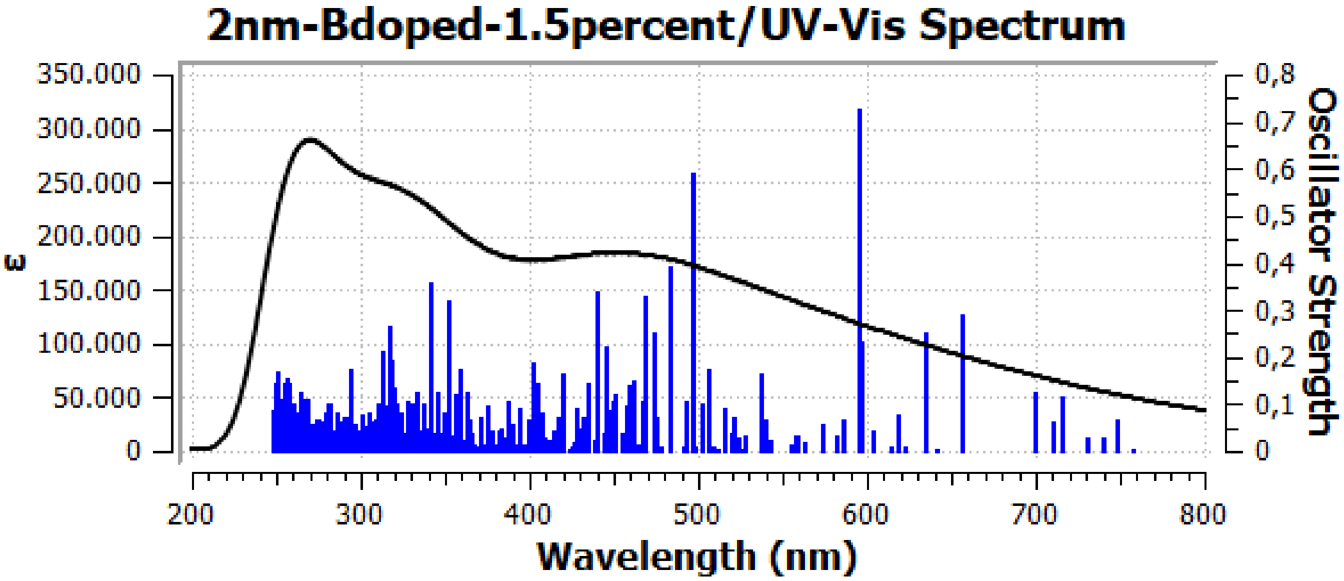

Figure S44. UV-Vis spectrum of 2 nm 1.5% B-doped graphene.

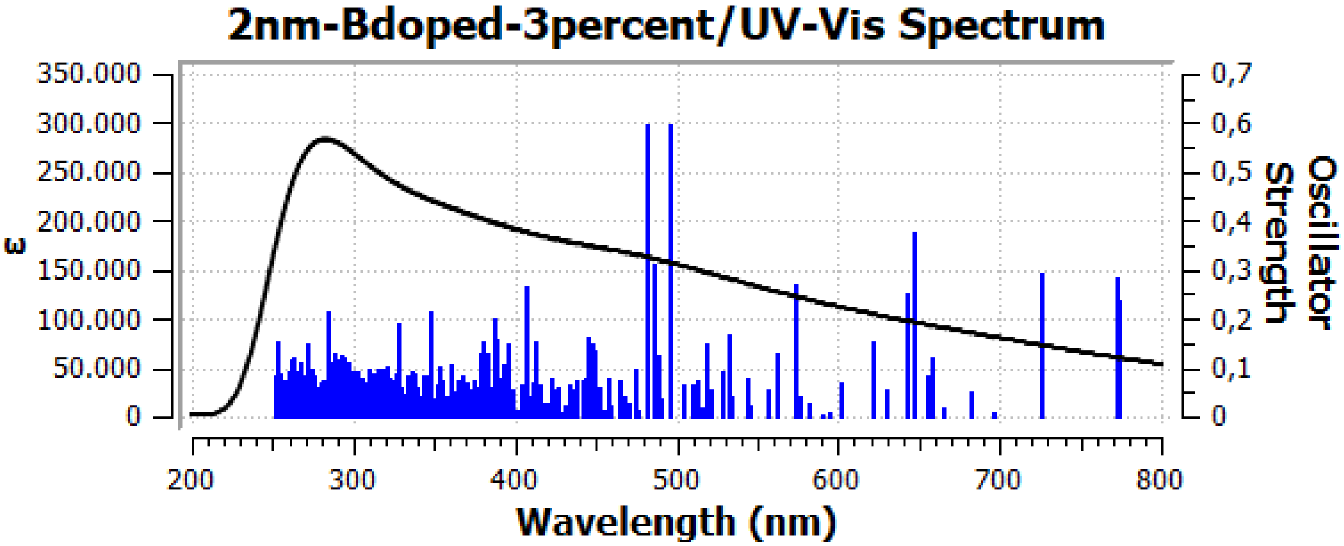

Figure S45. UV-Vis spectrum of 2 nm 3% B-doped graphene.

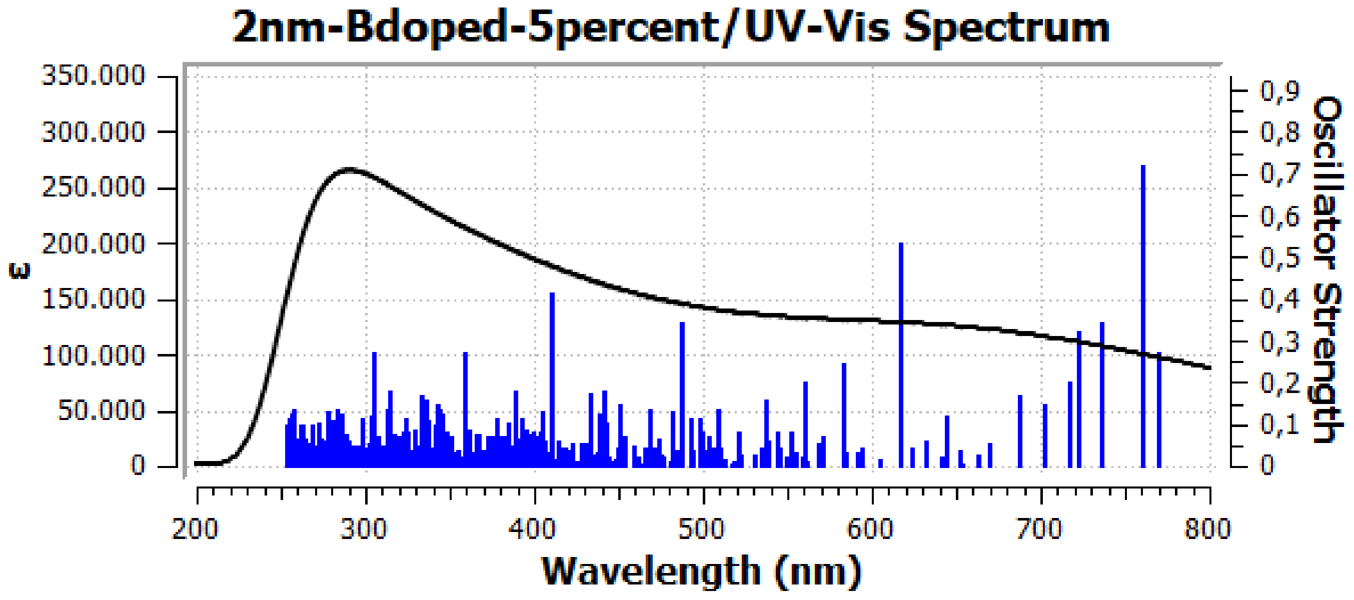

Figure S46. UV-Vis spectrum of 2 nm 5% B-doped graphene.

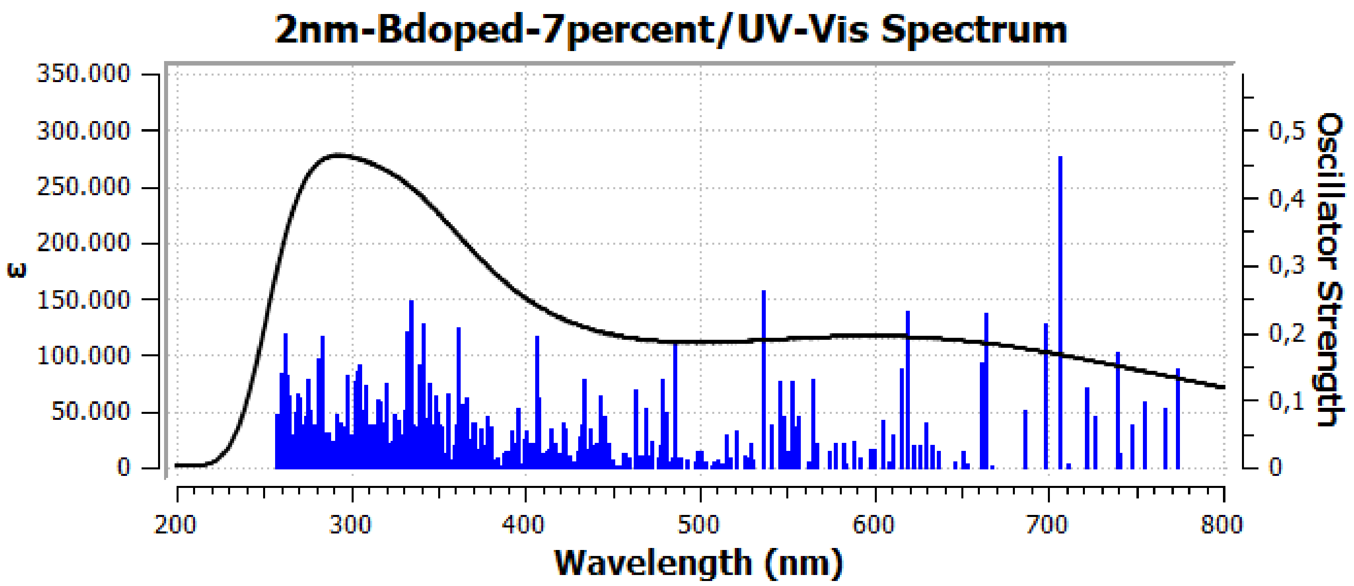

Figure S47. UV-Vis spectrum of 2 nm 7% B-doped graphene.

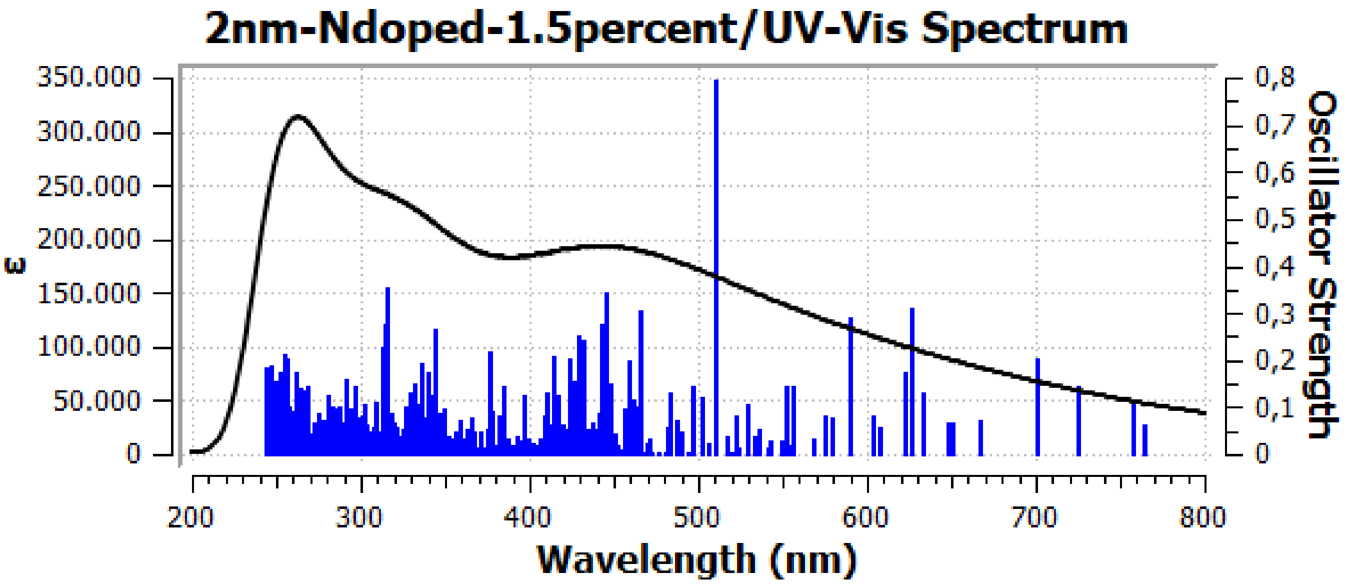

Figure S48. UV-Vis spectrum of 2 nm 1.5% N-doped graphene.

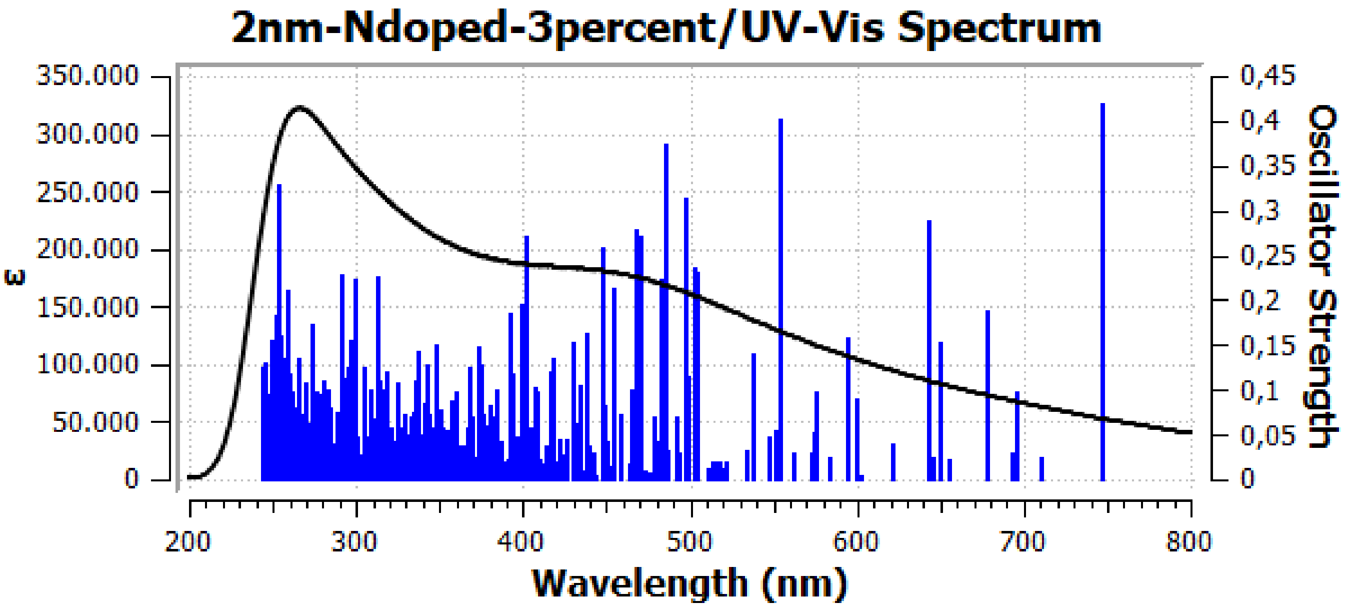

Figure S49. UV-Vis spectrum of 2 nm 3% N-doped graphene.

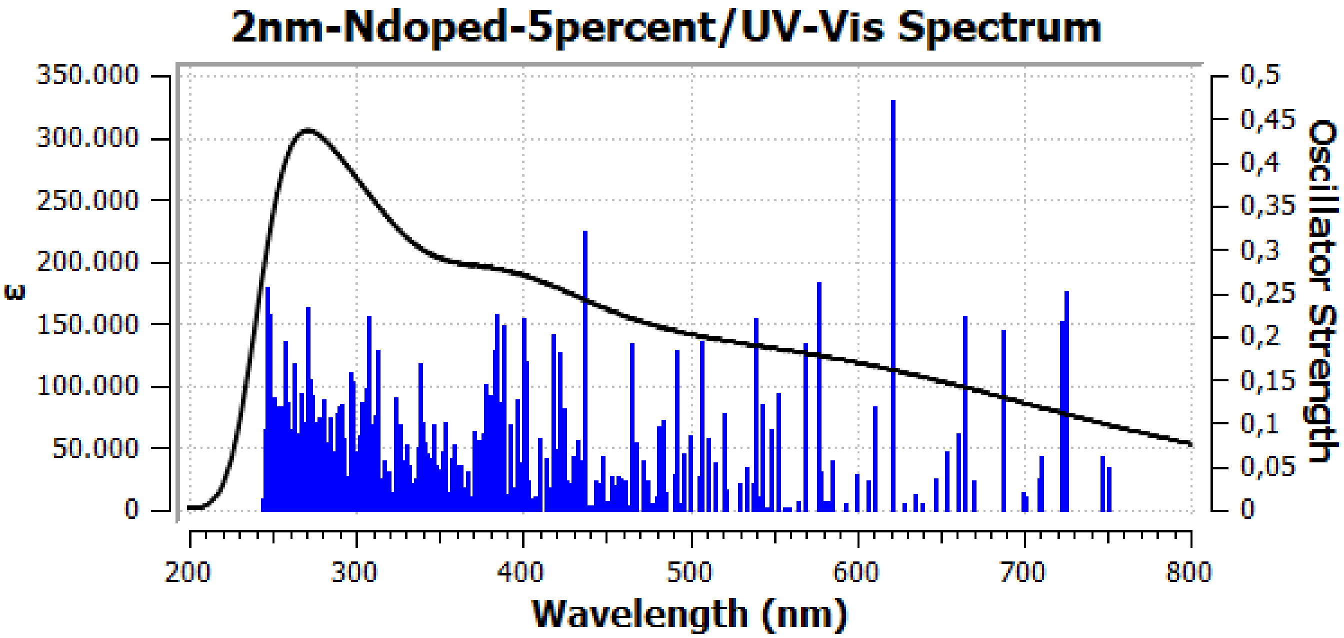

Figure S50. UV-Vis spectrum of 2 nm 5% N-doped graphene.

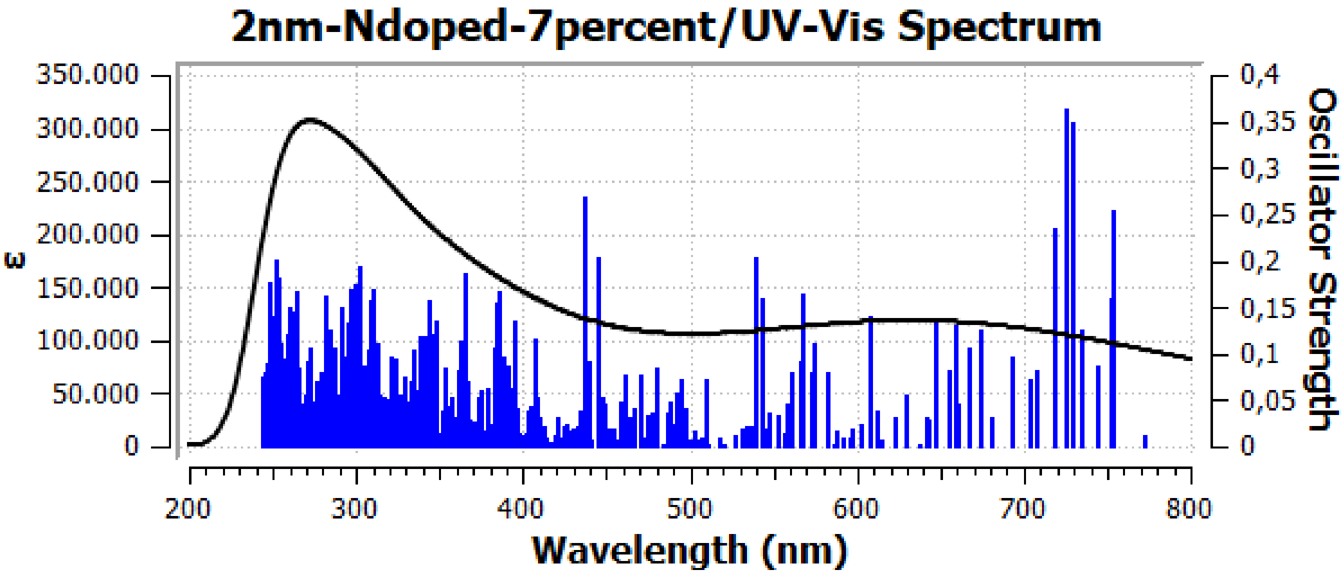

Figure S51. UV-Vis spectrum of 2 nm 7% N-doped graphene.

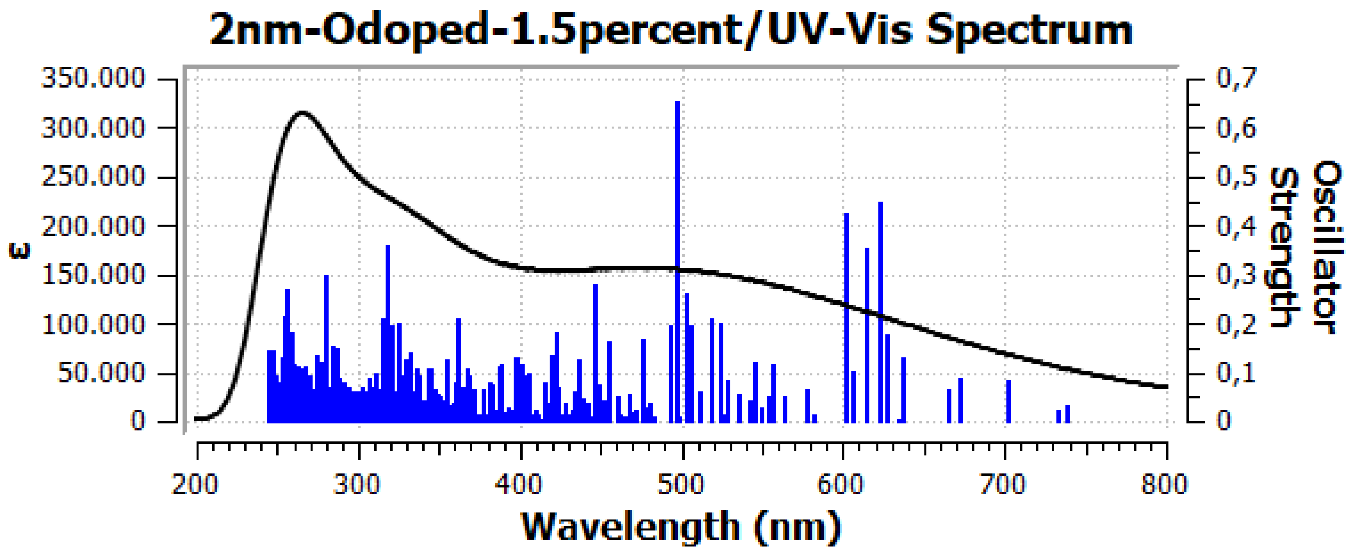

Figure S52. UV-Vis spectrum of 2 nm 1.5% O-doped graphene.

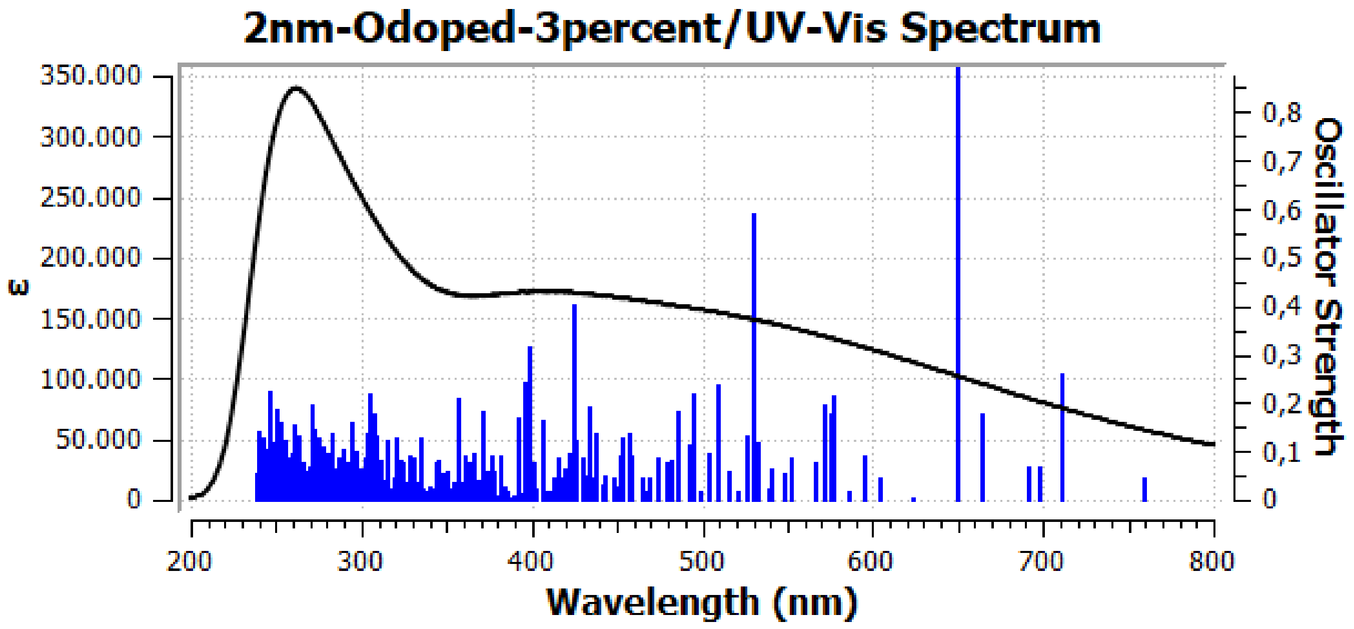

Figure S53. UV-Vis spectrum of 2 nm 3% O-doped graphene.

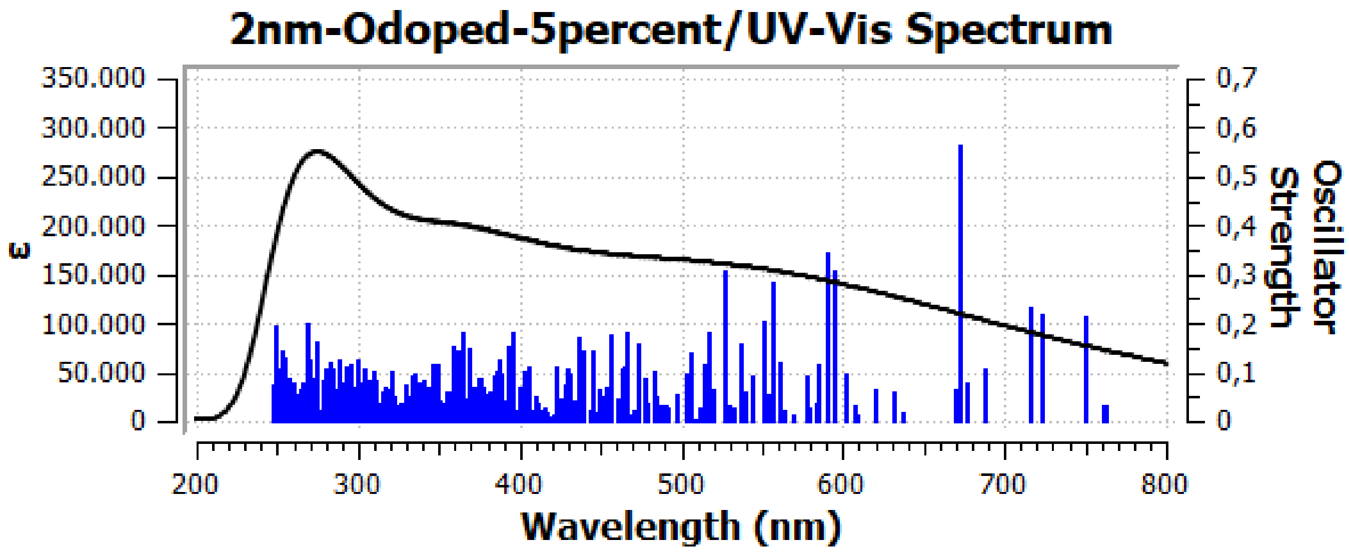

Figure S54. UV-Vis spectrum of 2 nm 5% O-doped graphene.

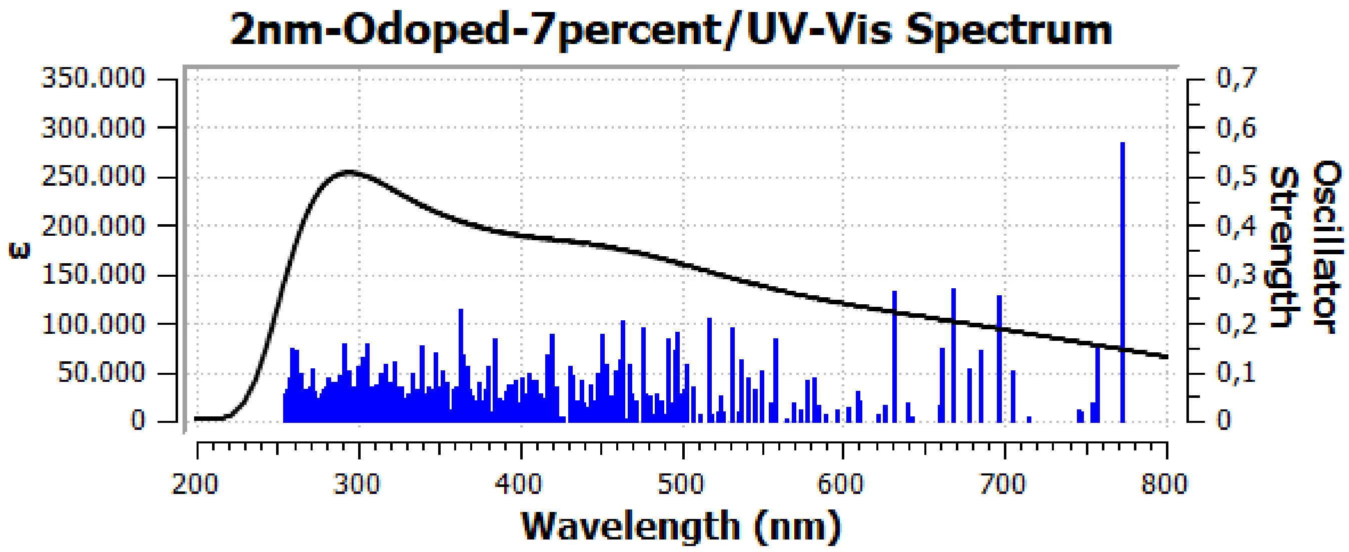

Figure S55. UV-Vis spectrum of 2 nm 7% O-doped graphene.

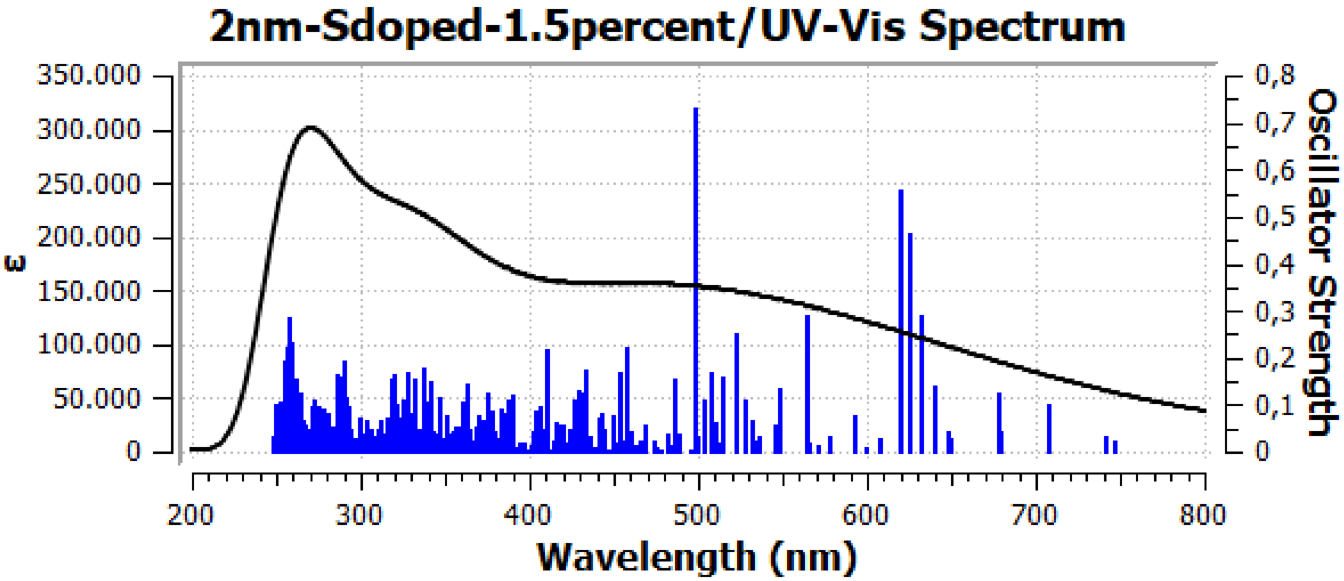

Figure S56. UV-Vis spectrum of 2 nm 1.5% S-doped graphene.

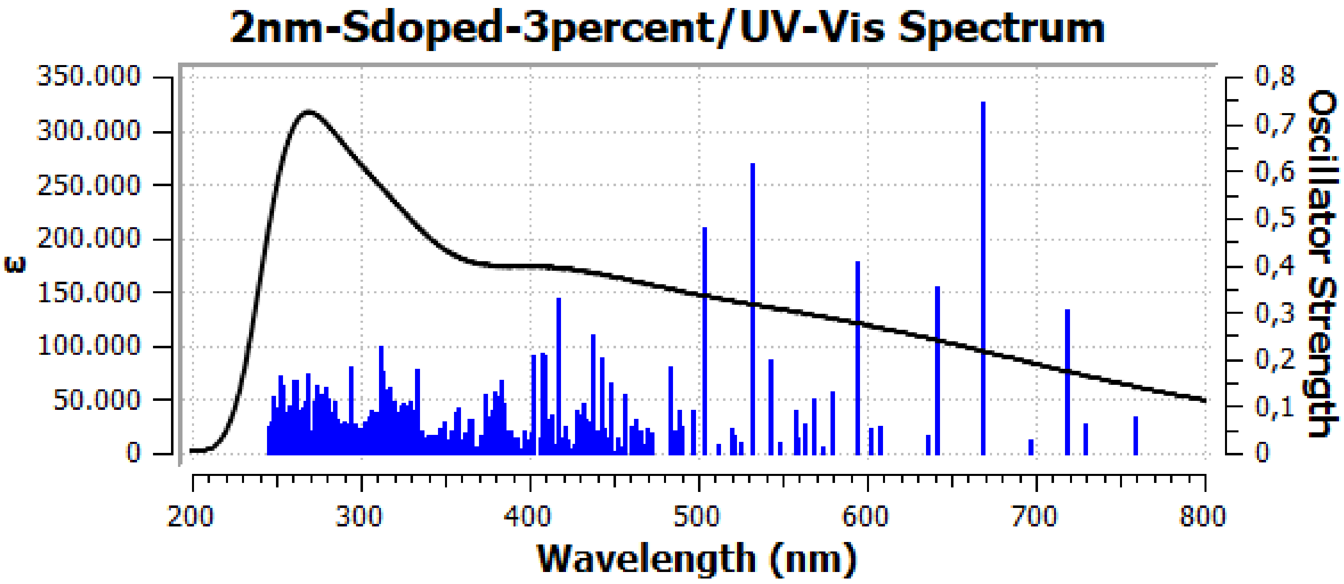

Figure S57. UV-Vis spectrum of 2 nm 3% S-doped graphene.

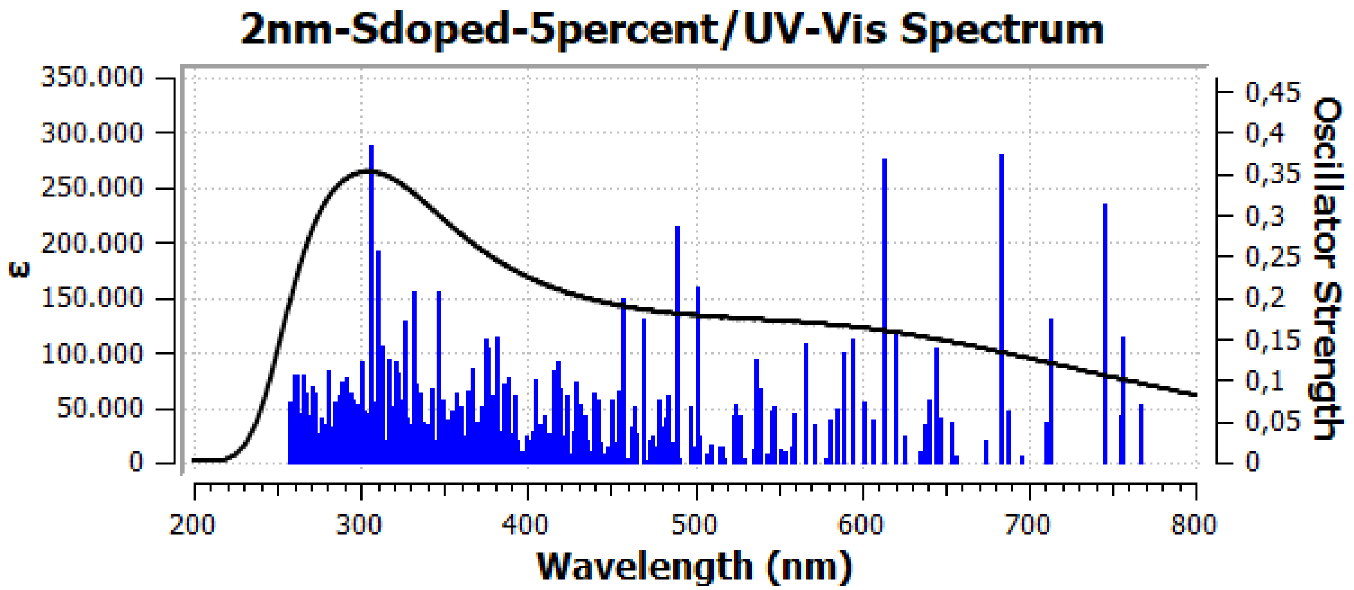

Figure S58. UV-Vis spectrum of 2 nm 5% S-doped graphene.

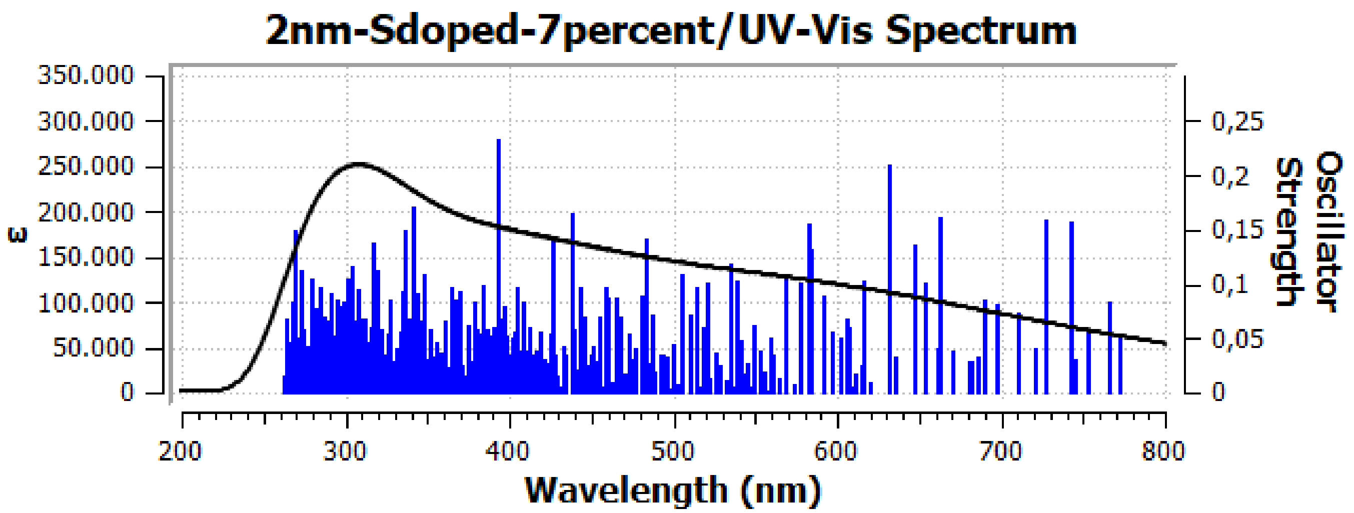

Figure S59. UV-Vis spectrum of 2 nm 7% S-doped graphene.

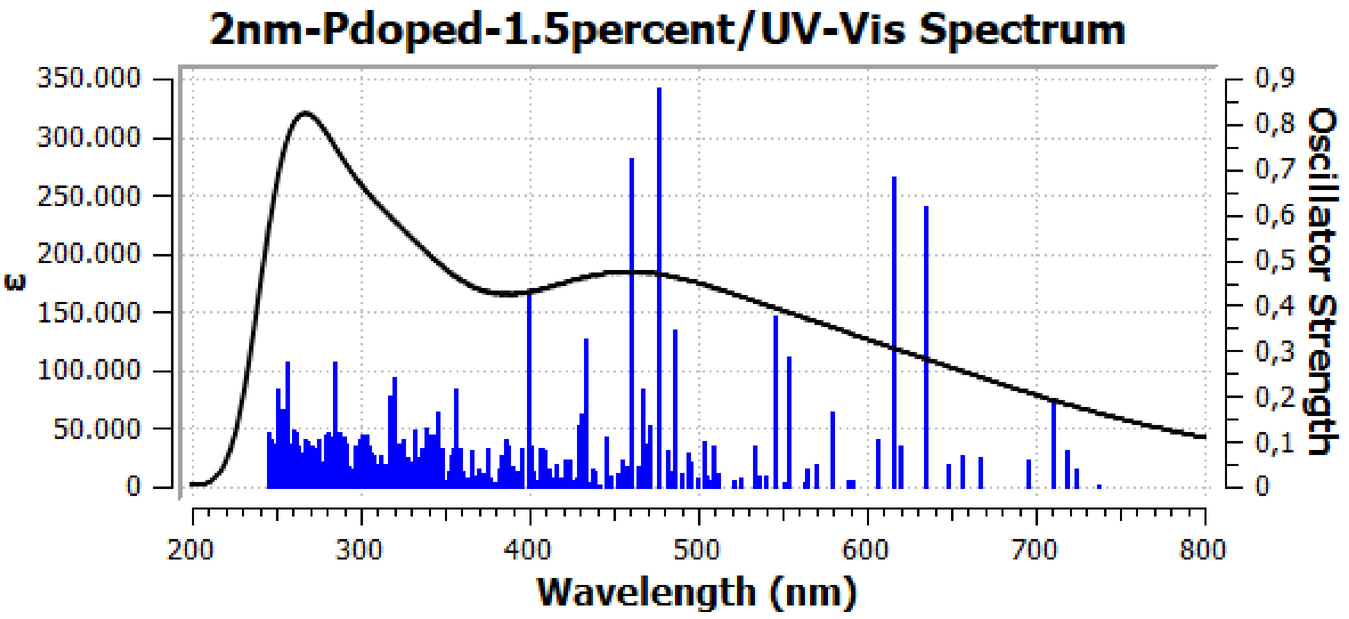

Figure S60. UV-Vis spectrum of 2 nm 1.5% P-doped graphene.

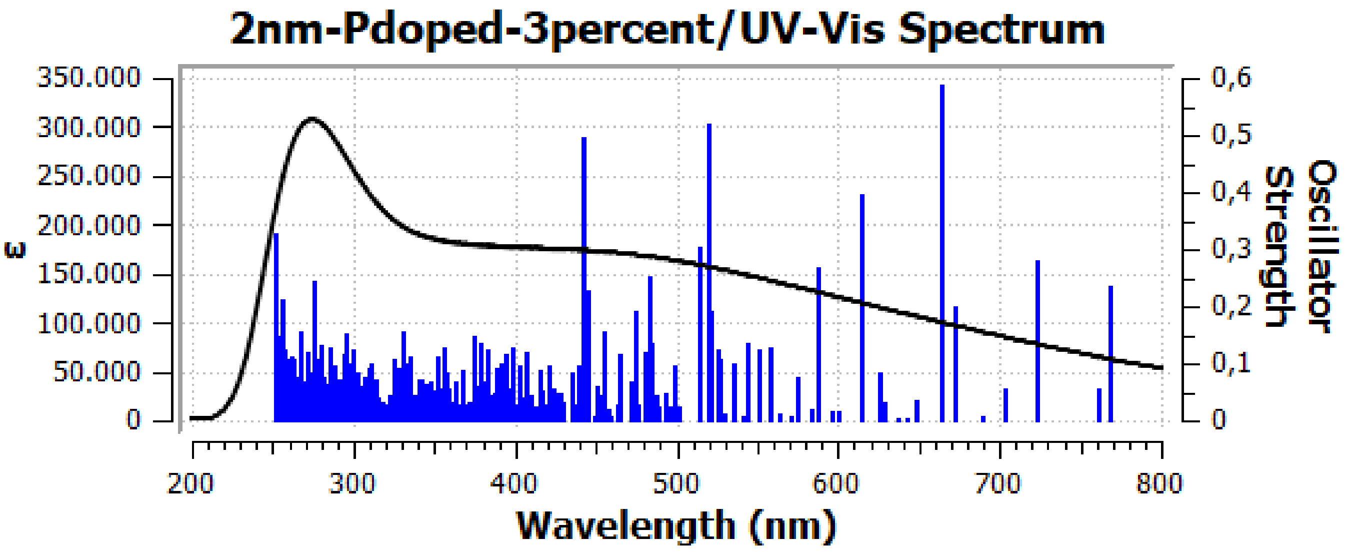

Figure S61. UV-Vis spectrum of 2 nm 3% P-doped graphene.

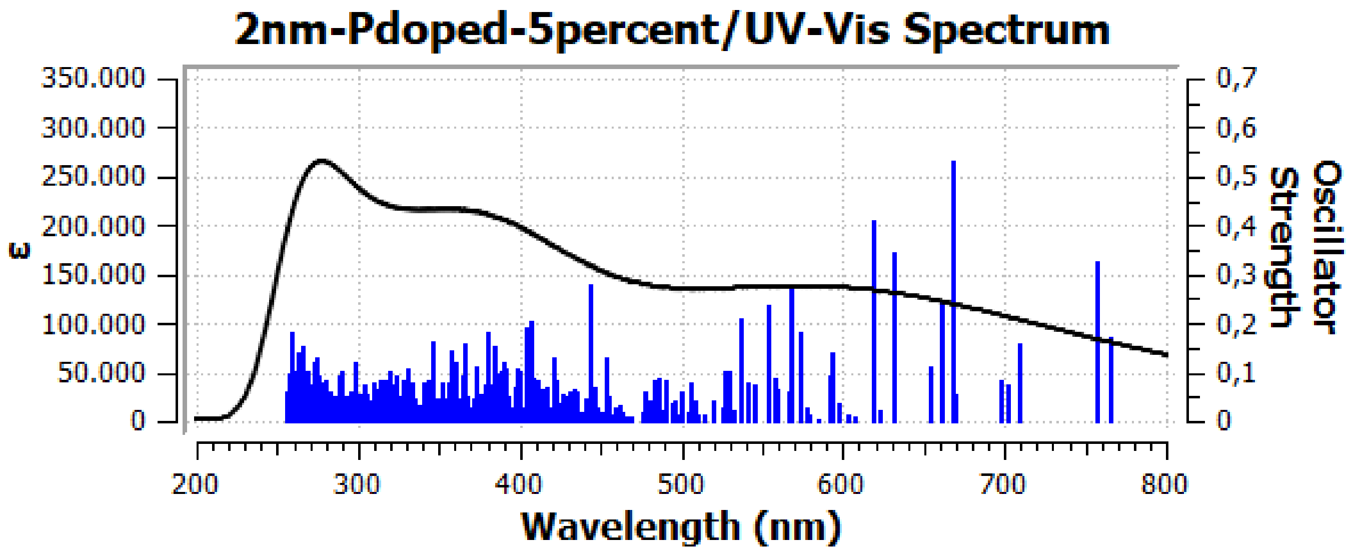

Figure S62. UV-Vis spectrum of 2 nm 5% P-doped graphene.

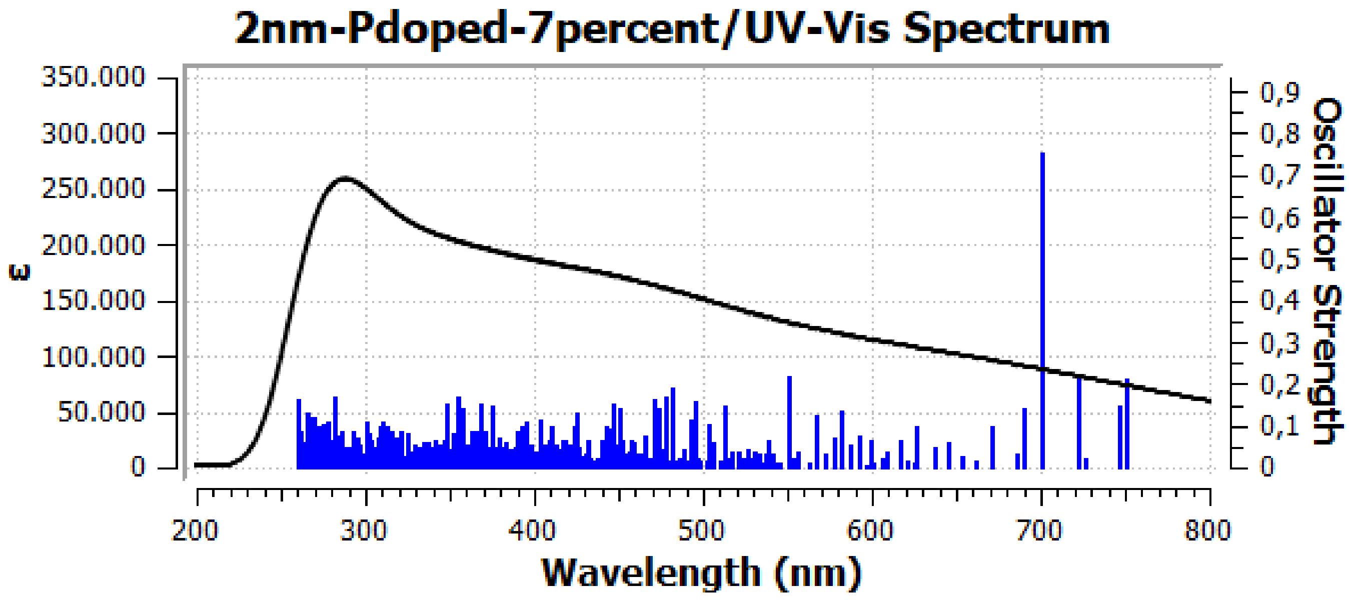

Figure S63. UV-Vis spectrum of 2 nm 7% P-doped graphene.
